# Two type I topoisomerases maintain DNA topology in human mitochondria

**DOI:** 10.1093/nar/gkac857

**Published:** 2022-10-10

**Authors:** Katja E Menger, James Chapman, Héctor Díaz-Maldonado, Mushtaq M Khazeem, Dasha Deen, Direnis Erdinc, John W Casement, Valeria Di Leo, Angela Pyle, Alejandro Rodríguez-Luis, Ian G Cowell, Maria Falkenberg, Caroline A Austin, Thomas J Nicholls

**Affiliations:** Wellcome Centre for Mitochondrial Research, Newcastle University, Newcastle upon Tyne NE2 4HH, UK; Biosciences Institute, Newcastle University, Newcastle upon Tyne NE2 4HH, UK; Wellcome Centre for Mitochondrial Research, Newcastle University, Newcastle upon Tyne NE2 4HH, UK; Biosciences Institute, Newcastle University, Newcastle upon Tyne NE2 4HH, UK; Department of Medical Biochemistry and Cell Biology, University of Gothenburg, PO Box 440, 405 30 Gothenburg, Sweden; Biosciences Institute, Newcastle University, Newcastle upon Tyne NE2 4HH, UK; Wellcome Centre for Mitochondrial Research, Newcastle University, Newcastle upon Tyne NE2 4HH, UK; Translational and Clinical Research Institute, Newcastle University, Newcastle upon Tyne NE2 4HH, UK; Department of Medical Biochemistry and Cell Biology, University of Gothenburg, PO Box 440, 405 30 Gothenburg, Sweden; Bioinformatics Support Unit, Faculty of Medical Sciences, Newcastle University, Newcastle upon Tyne, UK; Wellcome Centre for Mitochondrial Research, Newcastle University, Newcastle upon Tyne NE2 4HH, UK; Translational and Clinical Research Institute, Newcastle University, Newcastle upon Tyne NE2 4HH, UK; Wellcome Centre for Mitochondrial Research, Newcastle University, Newcastle upon Tyne NE2 4HH, UK; Translational and Clinical Research Institute, Newcastle University, Newcastle upon Tyne NE2 4HH, UK; Wellcome Centre for Mitochondrial Research, Newcastle University, Newcastle upon Tyne NE2 4HH, UK; Biosciences Institute, Newcastle University, Newcastle upon Tyne NE2 4HH, UK; Biosciences Institute, Newcastle University, Newcastle upon Tyne NE2 4HH, UK; Department of Medical Biochemistry and Cell Biology, University of Gothenburg, PO Box 440, 405 30 Gothenburg, Sweden; Biosciences Institute, Newcastle University, Newcastle upon Tyne NE2 4HH, UK; Wellcome Centre for Mitochondrial Research, Newcastle University, Newcastle upon Tyne NE2 4HH, UK; Biosciences Institute, Newcastle University, Newcastle upon Tyne NE2 4HH, UK

## Abstract

Genetic processes require the activity of multiple topoisomerases, essential enzymes that remove topological tension and intermolecular linkages in DNA. We have investigated the subcellular localisation and activity of the six human topoisomerases with a view to understanding the topological maintenance of human mitochondrial DNA. Our results indicate that mitochondria contain two topoisomerases, TOP1MT and TOP3A. Using molecular, genomic and biochemical methods we find that both proteins contribute to mtDNA replication, in addition to the decatenation role of TOP3A, and that TOP1MT is stimulated by mtSSB. Loss of TOP3A or TOP1MT also dysregulates mitochondrial gene expression, and both proteins promote transcription elongation *in vitro*. We find no evidence for TOP2 localisation to mitochondria, and TOP2B knockout does not affect mtDNA maintenance or expression. Our results suggest a division of labour between TOP3A and TOP1MT in mtDNA topology control that is required for the proper maintenance and expression of human mtDNA.

## INTRODUCTION

Mammalian cells contain two sources of DNA. While the vast majority of genetic material is contained within the nucleus, mitochondria contain their own small circular dsDNA genome called mitochondrial DNA (mtDNA). The mitochondrial genome is ancestrally bacterial and is typically present at thousands of copies per cell. It encodes only thirteen proteins, all of which are essential subunits of the respiratory chain complexes responsible for the majority of cellular energy transduction (1). Mitochondria possess machineries for DNA replication, transcription and translation that are largely distinct from those in the nucleus. Mitochondrial DNA replication occurs by an unusual asynchronous mechanism, with replication primers being synthesised by the mitochondrial RNA polymerase (1–3). Transcription in mitochondria is polycistronic, and produces almost genome-length RNAs that require post-transcriptional endonucleolytic processing (4). All of the estimated 300 proteins required for mtDNA maintenance and expression (5,6) are encoded in the nucleus, consisting of proteins homologous to bacterial, eukaryotic and bacteriophage proteins (7,8). Nuclear-encoded mitochondrial proteins are directed for mitochondrial import using targeting sequences, and are transported as linear polypeptides across the mitochondrial double membrane (9).

Transactions between proteins and DNA during DNA replication, gene expression, and DNA packaging disrupt the double helical structure of DNA. During transcription, a translocating RNA polymerase complex creates localised regions of overwinding (positive supercoiling) ahead of the transcription bubble and underwinding (negative supercoiling) behind it (10,11). During DNA replication, intertwines of replicating daughter DNA molecules can additionally form behind the replication fork, and must be removed in order for the replicated molecules to be segregated (12).

Excessive unresolved negative supercoiling can promote the formation of R-loops and other non-standard DNA structures that can threaten genome stability, while excessive positive supercoiling can inhibit the progression of DNA or RNA polymerase complexes (13).

Topological problems in DNA are solved by topoisomerases, a ubiquitous family of enzymes that untwist and untangle DNA. The human genome contains six topoisomerases. The type IB topoisomerases TOP1 and TOP1MT are swivelases that are able to relieve supercoiling during transcription and replication (14,15). TOP1 is an exclusively nuclear protein, while TOP1MT contains an N-terminal mitochondrial targeting sequence (MTS) that directs its import into mitochondria (16). TOP1MT binds to the mitochondrial RNA polymerase POLRMT (17), suggesting that TOP1MT acts as a swivel to regulate supercoiling during transcription in mitochondria (18,19).

Type IA and type IIA topoisomerases, on the other hand, utilise an enzyme-bridged strand-passage mechanism whereby a break is formed in a single- or double-stranded DNA molecule (respectively), then an intact DNA strand is passed through the break, and the break is resealed. This mechanism permits the removal of either intramolecular supercoils (if the broken and passaged strands are from the same DNA molecule) or the decatenation of intermolecular DNA linkages (if the broken and passaged strands are from different DNA molecules). In the eukaryotic nucleus, type IIA topoisomerases are the primary enzymes that act in the removal of chromosome interlinks (20,21), while in *Escherichia coli* both type IIA and type IA topoisomerases can contribute to decatenation (22–24). The transcript of the human type IA topoisomerase TOP3A contains two translation start sites, generating either a longer isoform bearing a mitochondrial targeting sequence or a shorter isoform that lacks this targeting sequence and is directed to the nucleus (25–27). The loss of mitochondrial TOP3A leads to the accumulation of catenated mtDNA replication products, indicating that TOP3A is required for decatenation of replicated mtDNA molecules (28,29). TOP3B possesses topoisomerase activity towards both DNA and RNA. Together with the Tudor domain protein TDRD3, TOP3B has been observed to localise to the nucleus and also to the cytosol (30,31), where it is involved in the translation of certain mRNAs (32).

TOP2A and TOP2B are homologous type IIA topoisomerases with essential roles in nuclear DNA maintenance. TOP2A is expressed only in proliferating cells (33,34), and is required for removing chromosome interlinks during anaphase (35–37). TOP2B is expressed in all cells (38) and is predominantly required for transcription regulation (33,34,39). In addition to these nuclear roles, several studies have proposed roles for TOP2 isoforms in the maintenance of mtDNA. Type II topoisomerase activities have been described in mitochondrial fractions (40–43), and later studies have suggested a mitochondrial localisation for either a truncated form of TOP2B (44), or full-length TOP2B and TOP2A (45,46). The exact roles and contributions of topoisomerases to maintaining mtDNA topology during mtDNA replication and transcription remain poorly understood (47).

In this work, we have assessed the localisation and activity of the six human topoisomerases with a focus on mitochondria and their contributions to mtDNA maintenance. Our results indicate that mitochondria possess one type IA topoisomerase, TOP3A, and one type IB topoisomerase, TOP1MT. Both enzymes contribute to the maintenance of mtDNA topology during DNA replication. TOP3A is also required for mitochondrial transcription, particularly of promoter-distal transcripts. TOP3A and TOP1MT show partial functional redundancy, and based on our results we propose a division of labour between TOP3A and TOP1MT during mtDNA replication and expression.

## MATERIALS AND METHODS

### Cell culture and transfection

HeLa cells and HEK cells were maintained in Dulbecco's modified Eagle's medium (DMEM, Gibco 31966012), SH-SY5Y cells were maintained in a 50:50 mixture of minimal essential medium (MEM, Gibco 31095029) and Ham's F-12 Nutrient Mixture (Gibco 21765029), K562 cells were maintained in Iscove's modified Dulbecco's medium (IMDM, Gibco 31980022), and Nalm6 cells were maintained in RPMI 1640 medium (Gibco 61870010). All media were supplemented with 10% foetal bovine serum (FBS, Sigma Aldrich F9665), 100 U/ml penicillin and 100 μg/ml streptomycin (Pen Strep, Gibco 15140122).

Transfections using siRNA were carried out using Lipofectamine RNAiMAX (Invitrogen). HeLa cells were passaged and transfected in suspension using 5 nM of each siRNA, and re-transfected after 72 h for a total of six days of transfection. All siRNA oligonucleotides were Silencer Select siRNAs (Ambion), and details are provided in Supplementary Table S1.

For transient transfections, HeLa cells were seeded at a density of 1 × 10^5^ cells per well onto glass coverslips in 6-well plates and left for 16 h prior to transfection. Plasmid transfections were performed for 24–48 h using Lipofectamine 3000 (Invitrogen L3000015), according to the manufacturer's instructions. For each transfection, 2500 ng of DNA was prepared in serum-free OptiMEM media (Thermo Fisher 31985070). Details of plasmid constructs are provided in the section ‘Cloning and DNA templates’.

### CRISPR-Cas9 knockout of TOP2B in SH-SY5Y, Nalm6 and K562

*TOP2B* exon 1 was targeted with a single guide RNA oligonucleotide (gRNA) (CGCGCCGCAGCCACCCGACT). This guide RNA was cloned into the plasmid vector pSpCas9 (BB)-2A-GFP (PX458) and the resulting construct was transfected into SH-SY5Y, K562 and Nalm6 cells by Nucleofection (Nucleofector II system, Amaxa, USA) using the Amaxa^®^ Cell Line Nucleofector® Kit T (Lonza, UK, cat. VCA-1002, for Nalm6 cells) or Amaxa® Cell Line Nucleofector® Kit V (Lonza, UK, cat. VCA-1003, for SH-SY5Y and K562 cells) according to the manufacturer's instructions. Cells were selected and sorted based on GFP expression as reported previously (48) as a single cell per well into 96-well plates and incubated at 37°C for 2–3 weeks until colonies formed. Colonies were expanded, and screening for TOP2B null clones was performed using genotyping and immunofluorescence as in (48).

pSpCas9(BB)-2A-GFP (PX458) was a gift from Feng Zhang (Addgene plasmid # 48138; http://n2t.net/addgene:48138; RRID:Addgene_48138) (49).

### Mitochondrial isolation from cultured cells

Mitochondria were isolated from ∼1.5 × 10^8^ cells by a differential centrifugation method adapted from (50). All steps were performed on ice. Cells were collected and washed twice with PBS by centrifugation at 300 *g* for 5 min. Cell pellets were weighed and resuspended in nine volumes (assuming a cell density of 1.25 g/ml) of hypotonic buffer (20 mM HEPES (pH 8), 5 mM KCl, 1.5 mM MgCl_2_, 2 mM DTT, 1 mg/ml BSA, 1 mM phenylmethylsulfonyl fluoride (PMSF) and 1× protease inhibitors (Thermo Scientific A32965)). After incubation on ice for 10 min, cells were homogenised by 10 strokes of a 15 ml glass Dounce homogeniser with tight-fitting pestle. A 5% sample was retained as the whole cell extract fraction. A two-thirds volume of 2.5× MSH buffer (525 mM mannitol, 175 mM sucrose, 20 mM HEPES (pH 8), 5 mM EDTA, 1 mg/ml BSA, 0.2 mM PMSF, 2 mM DTT and 1× protease inhibitors) was added and the lysate was centrifuged at 1600 *g* for 10 min. The pellet from this step was retained as the nuclear fraction, and the supernatant was centrifuged again at 1600 *g* for 10 min. The pellet was discarded, and the supernatant centrifuged at 10 000 *g* for 10 min. The supernatant from this step was retained as the cytosolic fraction, while the pellet was resuspended in 10 ml DNase buffer (210 mM mannitol, 70 mM sucrose, 20 mM HEPES (pH 8), 10 mM MgCl_2_, 2 mM EDTA, 1 mg/ml BSA, 1 mM PMSF and 1× proteinase inhibitors) and treated with 100 U DNase I (Ambion AM2222) for 1 h on a roller at 4°C. EDTA was added to a final concentration of 15 mM and the pellet was washed twice with 1× MSH (DNase buffer omitting MgCl_2_ and BSA). The pellet was then resuspended in 100 μl of 1× MSH and loaded onto a two-step 1.5/1 M sucrose gradient made up in gradient buffer (10 mM HEPES (pH 7.8), 5 mM EDTA, 2 mM DTT). Sucrose gradients were centrifuged at 117 000 *g*_max_ (33 000 rpm in a Beckman Coulter MLS50 rotor) and mitochondria recovered from the interface of the sucrose fractions. Four volumes of gradient buffer were added, mitochondria were washed and pelleted twice with 1× MSH, and then snap frozen in liquid nitrogen. Whole-cell extract and nuclear fractions (200 μl) were treated with 250 U Benzonase for 30 min at 37°C to remove genomic DNA prior to protein extraction.

For western blotting of TOP1MT in crude mitochondrial fractions, mtDNA extraction for 2DNAGE, analyses of mtDNA topology, and whole genome sequencing, the above protocol was followed until the DNase step.

### Proteinase K and digitonin treatments of isolated mitochondria

Sucrose gradient purified mitochondria were resuspended in 1× MSH without PMSF or proteinase inhibitors and divided into three equal volumes. Samples were either untreated (mitochondrial fraction), or treated with 25 μg/ml proteinase K at 37°C for 30 min in the absence or presence of 1% (v/v) Triton X-100. PMSF was added to proteinase-treated samples to a final concentration of 5 mM, then mitochondria were pelleted and washed twice in 1× MSH and snap frozen. For mitoplast preparations, equal volumes of sucrose gradient purified mitochondria were resuspended in 1× MSH buffer without PMSF or proteinase inhibitors, and digitonin was added to the indicated concentrations in a final volume of 500 μl. Samples were incubated on ice for 10 min and then mitochondria were pelleted at 15 000 *g* for 15 min. Samples were then resuspended in 1× MSH buffer and divided into two, and one set of samples was treated with 25 μg/ml proteinase K for 30 min at 37°C, then washed and frozen as above.

### Animals

C57BL6/J mice were housed in single-sex cages at 20 ± 2°C with a 12 h light/dark cycle. Animal experiments were conducted in compliance with the UK Home Office (PPL P76987201) and the Newcastle University Animal Welfare Ethical Review Board (AWERB 425).

### Mitochondrial isolation from mouse tissues

Mitochondria were isolated from fresh mouse liver and spleen tissue according to the method of (51,52). Briefly, tissue was washed in ice-cold extraction buffer (250 mM sucrose, 250 mM mannitol, 25 mM HEPES, 10 mM KCl, 0.25 mM EDTA, 10 mM EGTA, 1.5 mM MgCl_2_, 1 mM DTT, 0.1% (w/v) BSA, 1× protease inhibitors, pH 7.4) then cut into small pieces using a fresh scalpel. The tissue was transferred into a 15 ml glass Dounce homogeniser in 10 ml of extraction buffer and disrupted with ten strokes of a loose-fitting pestle followed by ten strokes of a tight-fitting pestle. The volume was adjusted to 20 ml with extraction buffer, and a sample retained as the whole cell extract. The remaining sample was centrifuged at 700 *g* for 10 min at 4°C. The pellet was retained as the nuclear fraction, and the supernatant was centrifuged at 10 000 *g* for 15 min at 4°C. A sample of the supernatant was retained as the cytosolic sample. The pellet was resuspended in 10 ml of DNase buffer, and DNase treatment and sucrose gradient centrifugation were carried out as described in the section ‘Mitochondrial isolation from cultured cells’.

### Cloning and DNA templates

The full-length cDNA sequences for TOP1 (Genscript NM_003286.4), TOP1MT (Genscript NM_052963), TOP3A (Genscript NM_004618.5) and TOP3B (Genscript NM_003935.4) were amplified (omitting the stop codon), incorporating 5′ KpnI and 3′ XhoI restriction sites in the primer sequences. These were then cloned into pcDNA5/FRT/TO in frame with eGFP, which was cloned between the XhoI and ApaI restriction sites. To create the Δ25-TOP3A-eGFP construct, the TOP3A cDNA sequence was amplified from M26 and cloned as above.

For *in vitro* assays, the mtDNA region containing LSP (nt. 363 to nt. 498, NC_012920) followed by a random DNA sequence upstream of the promoter, were obtained from Eurofins Genomics in the form of linear dsDNA (GeneStrands). LSP-containing DNA fragments were cloned into pUC19 between HindIII and PstI restriction sites (for LSP-A) and PstI and BamHI restriction sites (for LSP-B). In order to confirm whether both cloned promoters have equivalent transcription efficiency, the plasmid was linearised with HindIII and BamHI for use as a template for *in vitro* transcription reactions. The dual promoter template was used in a supercoiled conformation when the effect of topoisomerases in an *in vitro* transcription system was assayed. For relaxation experiments, a supercoiled pUC18 plasmid containing the LSP region (positions 1–477 in mtDNA) was used.

### Immunofluorescence

For HeLa cells, fixation of cells transfected with topoisomerase-eGFP constructs was performed using 4% (v/v) paraformaldehyde (PFA) with 0.02% (w/v) EM Grade L-Glutaraldehyde (2BScientific). For antibody labelling of TOP2A and TOP2B, optimised fixation was performed using 4% (v/v) PFA and 8% (v/v) PFA respectively. In each case fixation was performed for 10 minutes at room temperature. Permeabilisation was performed in 0.5% (v/v) Triton X‐100 in PBS for 15 min, followed by blocking in 5% (w/v) BSA in PBS for 1 h. Coverslips were then incubated with primary antibodies overnight in a humidified chamber at 4°C. The following day the coverslips were washed extensively with PBS prior to incubation with secondary antibodies (at a dilution of 1:500) for 2 h at room temperature. The details of all primary and secondary antibodies used are provided in Supplementary Table S2. Following incubation, the coverslips were washed in PBS and then incubated with DAPI solution (5 μg/ml) for 5 min. Coverslips were then washed extensively with PBS prior to mounting on slides using ProLong Glass Antifade Mountant (Invitrogen, Cat no. P36980). Slides were left to dry for at least 12 h prior to imaging.

For detection of *de novo* transcription products in HeLa cells, cells were pulsed with 2.5 mM BrU (Merck Millipore, 850187–1G) for 1 h prior to fixation using 4% (v/v) PFA with 0.02% (w/v) EM Grade l-glutaraldehyde as described above. Permeabilisation, blocking, labelling and mounting were performed as described earlier for HeLa cells. Incorporated BrU was detected using a primary antibody against BrdU and co-labelled with TOM20. Secondary antibodies were used at a dilution of 1:200 for 2 h at room temperature.

### Microscopy

For HeLa cells, Airyscan images were acquired on a Zeiss LSM800 with Airyscan microscope using a 63× 1.4 NA objective. The 405, 488 and 640 nm laser lines were used for image acquisition in conjunction with refractive index‐matched immersion oil (Zeiss). Airyscan processing of the raw images was performed using the Airyscan processing function in the ZEN software.

Following image acquisition and subsequent Airyscan processing of HeLa cells following staining of topoisomerases, images were analysed using the object analyser function in Huygens Essential deconvolution software (Scientific Volume Imaging). Specifically, the degree of co-localisation between the topoisomerase signal (or H3 and ATP5I controls) and either the DAPI or TOM20 signal for each cell was measured using the co-localisation analyser tool.

BrU-labelled HeLa cells were imaged by three-dimensional (3D) STED nanoscopy using a Leica TCS SP8 gSTED 3× microscope (Leica Microsystems) equipped with white light lasers. Images were acquired using a HC PL APO 100×/1.40 Oil STED WHITE objective and a voxel size of (10-20) × (10-20) × 100 nm (*xyz*) nm was used. The fluorophore Alexa Fluor 594 was excited at 590 nm and Atto647N at 646 nm using a 775 nm laser. Following acquisition, images were deconvolved using Huygens Essential deconvolution software and the object analyser tool was used to determine the number, size and intensity of BrU foci.

### RNA-seq and analysis

RNA extraction was performed using an RNeasy Mini kit (Qiagen, USA) according to the manufacturer's instructions. The total RNA concentration and RNA integrity number (RIN) of samples were determined using an Agilent 2100 Bioanalyzer System (Agilent Technologies, Germany). For SH-SY5Y WT and TOP2B knockout cells, a TruSeq Stranded mRNA kit (Illumina) was used to select polyA+ RNA molecules and libraries were constructed using the High Sample (HS) Protocol of the TruSeq^®^Stranded mRNA Sample Preparation Guide (Illumina). Sequencing (75 bp single end) was performed on the Illumina NextSeq 500 platform.

For TOP1MT, TOP3A and TOP1MT + TOP3A siRNA-treated cells, RNA samples were processed by the Oxford Genomics Centre. Briefly, samples were depleted using NEB human probes. The mRNA fraction was selected from total RNA and converted to cDNA. cDNA was end-repaired, A-tailed and adapter ligated. Sequencing (150 bp paired end) was performed on the Illumina NovaSeq6000 platform. Quality checks of raw RNASeq data were made using FastQC (v.0.11.9) and FastQ screen (v0.14.1) (53); the quality of the aligned data was checked using alignment statistics from HISAT2 (v2.2.1) (54), featureCounts (v2.0.0) (55) and QualiMap (v2.2.1) (56), and the threshold for data quality were met. The count estimates for the transcripts were obtained using Salmon (v1.3.0) (57) with sequence, GC and positional bias corrections. The genome version used was *Homo sapiens* GRCh38; and quantification was carried out using only coding transcripts. The counts were summarised at gene level using tximport (v.3.13) (58). The annotation used for RNASeq differential expression (DE) analysis was Homo_sapiens. GRCh38.101, protein coding genes only. DeSEQ2 (v.1.30.1) was used for differential expression (DE) modelling. The DE modelling was performed with or without minimum effect size specified as fold change >2 and at FDR <0.05. Reads mapped to mitochondrial DNA were extracted and split according to strand origin using samtools view (v1.12) (59,60). Nucleotide coverage was extracted for strand specific reads using samtools depth -r MT -a -d 0 -q 30. Counts were normalised at each nucleotide position to reads per million aligned. For depth coverage, reads were subsequently normalised to counts at 562 or 406 respectively, depending on strand origin and the log2(fold change) relative to ctrl samples was calculated.

### Whole genome sequencing

Crude mitochondria were isolated from siRNA-treated HeLa cells as described in ‘Mitochondrial isolation from cultured cells’, and DNA was extracted from mitochondrial pellets using a DNeasy Blood and Tissue Kit (Qiagen) according to the manufacturer's instructions. Isolated DNA (500 ng) was used for library preparation and sequencing, which were carried out by the Newcastle University Genomics Core Facility. Libraries were prepared using an Illumina DNA PCR-Free Prep kit, and sequencing was performed on an Illumina Novaseq6000 at 30× median genome coverage.

The quality of sequencing results was assessed using FastQC and MultiQC. Sequences were aligned to the human reference genome with bowtie2 (v.2.3.4, GRCh38_noalt) (61). Aligned reads were sorted and indexed using SAMtools (v.1.12, samtools sort -O bam, samtools index). In order to account for reads crossing the origin of mtDNA, reads mapped to the mitochondrial genome as well as unmapped reads were extracted and realigned to a mitochondrial genome which was shifted by 8,000 nt (62). Realigned reads were sorted and indexed as above. Coverage at nucleotide positions for the mitochondrial genome was determined using samtools depth -r NC_012920.1 -a -d 0 -q 30 for both original mitochondrial reads as well as realigned reads. Information for depth coverage was spliced together from both files with nucleotide coverage for position 4000–12 000 being obtained from the original aligned reads, while positions 1–3999 and 12 001–16 569 were extracted from the realigned reads. Further processing was performed in R. Nucleotide counts were normalised to counts at nt. 1 within each sample (control, TOP3A, TOP1MT and TOP3A + TOP1MT siRNA-treated cells), and the topoisomerase siRNA-treated samples were subsequently normalised at each nucleotide position to the control sample. Moving averages with a 21 bp window were calculated (using the fpp3 package) and the depth coverage was plotted against the mtDNA position. In addition, overall depth was plotted for each sample separately.

### Western blotting

Cell pellets were resuspended in lysis buffer (50 mM Tris–HCl (pH 7.4), 150 mM NaCl, 1 mM EDTA, 1% (v/v) Triton X-100, 1× protease inhibitors), incubated at 4°C on a roller for 30 min, then centrifuged for 3 min at 11 000 *g* at 4°C and the supernatant retained. Equal amounts of protein (determined by BCA assay) were separated on 4–20% Criterion TGX SDS-PAGE gels (Bio-Rad) and electroblotted onto nitrocellulose membranes. Membranes were blocked in 5% (w/v) milk (Marvel) in PBS at room temperature for 1 h. Primary antibodies were incubated in 5% (w/v) milk in PBS overnight on a roller at 4°C. Membranes were washed three times for 10 min with 0.1% (v/v) Triton X-100 in PBS, then incubated with the corresponding secondary antibodies in 5% (w/v) milk in PBS for 1 h at room temperature. Membranes were washed a further three times as above, then developed using Pierce ECL (Thermo Scientific 32109), SuperSignal West Pico PLUS (Thermo Scientific 34580), or SuperSignal West Femto (Thermo Scientific 34096). Details of all antibodies used are provided in Supplementary Table S2.

### Quantitative PCR

DNA was isolated from 0.5–1 × 10^6^ cells using a DNeasy Blood and Tissue DNA isolation kit (Qiagen 69504) according to manufacturers' protocol. DNA samples were diluted with water to 10 ng/μl. Quantitative PCR was performed for the nuclear target *B2M* as well as the mtDNA target *MT-ND1* using 2× Taqman universal PCR master mix (Applied Biosystems 4305719). Reactions (20 μl) contained 50 ng of DNA, 300 nM forward and reverse primers and 100 nM probe. Oligo sequences used are provided in Supplementary Table S3.

A standard curve amplifying both the *MT-ND1* and *B2M* targets from a single plasmid was run alongside each experiment. Samples were run in technical triplicates and at least three biological replicates. qPCR was performed on a StepOnePlus™ Real-Time PCR System using the following cycling conditions: 2 min at 50°C, 10 min at 95°C, then 40 cycles of 15 s at 95°C and 1 min at 60°C (63).

Obvious outliers were removed from the data set prior to analysis and the standard curve was used to determine relative levels of *B2M* and *MT-ND1* within the sample. Relative mtDNA copy number was calculated as levels of *MT-ND1/B2M* and normalised to the levels in control transfected samples. Error bars were calculated as the standard error of the mean from the ratio of *MT-ND1/B2M* and subsequently transformed into percentages according to the normalised ratios.

### Analysis of mtDNA topology

Crude mitochondria were first isolated from cells as described in ‘Cell Fractionation’. Mitochondria were resuspended in lysis buffer (75 mM NaCl, 50 mM EDTA, 20 mM HEPES (pH 7.8), 0.5% (w/v) SDS, 0.2 mg/ml proteinase K) then DNA was isolated by sequential phenol and chloroform extractions, precipitated using isopropanol and resuspended in TE (pH 8). DNA samples (2 μg) were loaded onto 0.4% (w/v) agarose gels without ethidium bromide and separated at 35 V for 22 h at room temperature. Gels were sequentially incubated in depurination buffer (0.25 M HCl, 20 min), denaturation buffer (0.5 M NaOH, 1.5 M NaCl, 2 × 10 min) and neutralisation buffer (0.5 M Tris–HCl (pH 7.4), 1.5 M NaCl, 2 × 10 min), then blotted overnight onto nylon membrane. Membranes were UV crosslinked at 1200 mJ/cm^2^. Probes were made by radiolabelling PCR products using a Prime-It II kit (Agilent 300385) with α-^32^P dCTP (3000 Ci/mmol, Hartmann Analytic GmbH). Primers for probe synthesis are provided in Supplementary Table S3. Membranes were probed overnight at 60°C in hybridisation buffer (0.25 M phosphate buffer, 7% (w/v) SDS), then washed three times for 20 min with 1× SSC (150 mM NaCl, 15 mM sodium citrate (pH 7.0)) with 0.1% (w/v) SDS. Membranes were exposed to a storage phosphor screen and imaged using a Typhoon FLA 7000 (GE Healthcare).

### Two-dimensional neutral agarose gel electrophoresis (2DNAGE)

Two-dimensional neutral agarose gel electrophoresis was carried out according to the method of (64). DNA was extracted from isolated mitochondria as described in ‘Analysis of mtDNA topology’ above. 5–10 μg of DNA was restricted using 20 U of HincII in a 400 μl reaction, then ethanol precipitated and resuspended in TE (pH 8). DNA was loaded onto first-dimension 0.4% (w/v) agarose gels without ethidium bromide and separated at 27 V for 18 h at room temperature. DNA-containing lanes were then cut from the gel, rotated 90° counter-clockwise and placed into a new tank. Molten 1% (w/v) agarose containing 500 ng/ml ethidium bromide was cast around the gel slices, and second-dimension gels were run at a constant 260 mA for 6 h at 4°C. Gels were transferred, hybridised and imaged as described above.

### Northern blotting

Total RNA was extracted from siRNA-treated cells using an RNeasy Mini kit (Qiagen), and RNA concentrations determined using a Nanodrop spectrophotometer. RNA was mixed with an equal volume of 2× RNA loading buffer (20 mM MOPS, 8 mM sodium acetate, 1 mM EDTA, 20% (v/v) glycerol, 6.5% (v/v) formaldehyde, 50% (v/v) formamide, 10 μg/ml ethidium bromide, 0.05% (w/v) bromophenol blue and 0.05% (w/v) xylene cyanol) and separated on 1.2% (w/v) denaturing agarose gels in 1× NorthernMax (Thermo Scientific) running buffer. Gels were washed in deionised water, blotted onto nylon membranes and then UV crosslinked at 1200 mJ/cm^2^. Probes were made by radiolabelling PCR products using a Prime-It II kit (Agilent 300385) using α-^32^P dCTP (3000 Ci/mmol, Hartmann Analytic GmbH), except for the 5.8S rRNA loading control, which was end-labelled with γ-^32^P ATP (3000 Ci/mmol, Hartmann Analytic GmbH) using T4 polynucleotide kinase (NEB). Primers for probe synthesis are provided in Supplementary Table S3. Membranes were probed overnight at 60°C (40°C for the 5.8S rRNA oligo probe) in hybridisation buffer (0.25 M phosphate buffer, 7% (w/v) SDS), then washed three times for 20 min with 1× SSC (150 mM NaCl, 15 mM sodium citrate (pH 7.0)) with 0.1% (w/v) SDS. Membranes were exposed to a storage phosphor screen and imaged using a Typhoon FLA 7000 (GE Healthcare). For analysis, images were imported into ImageLab software (version 6.0.1 build 34, standard edition, Bio-Rad Laboratories). Lanes and bands were manually identified and the background adjusted across all lanes per northern blot. The adjusted band volume was normalised to the corresponding adjusted band volume of the loading control 5.8S rRNA (RNA of interest/5.8S rRNA) and these values were normalised to the negative control siRNA samples, set as 1. One-way ANOVA was used to determine statistically significant differences between treatments.

### Protein purification

Recombinant mitochondrial RNA polymerase POLRMT (65), transcription factors TFB2M, TFAM (66,67), and TEFM (68), TOP1MT (66) and mtSSB (69) were expressed and purified according to previously described protocols. TOP3A (UniProt: Q13472, residues 16–934) bearing an additional N-terminal TEV-cleavable 6× His tag, was expressed in baculovirus-infected *Spodoptera frugiperda* Sf9 cells. Cell lysis was carried out in 20 mM Tris–HCl (pH 8), 500 mM NaCl, 10 mM β-mercaptoethanol and 1× proteinase inhibitors with a 1× freeze-thaw cycle using liquid nitrogen. The lysate was centrifuged at 20 000 rpm for 45 min at 4°C using a Sorvall Surespin 630 rotor to remove cell debris. The supernatant was applied to His-Select Nickel Affinity Gel (Sigma-Aldrich) equilibrated with Nickel buffer (25 mM HEPES (pH 7), 400 mM NaCl, 10% glycerol, 10 mM β-mercaptoethanol) containing 5 mM imidazole, then washed with Nickel buffer containing 10 mM imidazole and eluted with Nickel buffer containing 250 mM imidazole. The eluted protein was dialysed in 1 l of Nickel buffer overnight with TEV protease at 4°C. This was then loaded again onto His-Select Nickel Affinity Gel and the flow through was collected. The protein was further purified over HiTrap Heparin HP (GE Healthcare Life Sciences) equilibrated with Buffer A (25 mM HEPES (pH 7), 200 mM NaCl, 10% glycerol, 1 mM DTT, 0.5 mM EDTA (pH 8), 1 × proteinase inhibitors) and eluted with a linear gradient of Buffer B (25 mM HEPES (pH 7), 1.2 M NaCl, 10% glycerol, 1 mM DTT, 0.5 mM EDTA (pH 8), 1× proteinase inhibitors). The eluate was then loaded onto a Superdex 200 16/600 gel filtration column (GE Healthcare Life Sciences) and eluted with Buffer C (25 mM HEPES (pH 7), 400 mM NaCl, 10% glycerol, 1 mM DTT, 0.5 mM EDTA (pH 8), 1× proteinase inhibitors). Finally, the protein was loaded onto HiTrap SP HP (GE Healthcare Life Sciences) equilibrated with Buffer A and eluted with Buffer B as a linear gradient.

### *In vitro* transcription assays

*In vitro* transcription time-course experiments were performed in a reaction buffer consisting of 25 mM Tris–HCl pH 8.0, 10 mM MgCl_2_, 64 mM NaCl, 100 μg/ml BSA, 1 mM DTT, 400 μM ATP, 150 μM GTP, 150 μM CTP, 10 μM UTP, 0.02 μM α-^32^P UTP (3000 Ci/mmol, Hartmann Analytic GmbH) and 4 U RNase inhibitor Murine (New England Biolabs). 4 nM of DNA template, 20 nM POLRMT, 30 nM TFB2M, 5 nM TEFM and 280 nM TFAM were added to the master-mix. Where topoisomerases are added, reactions were supplemented with either 10 nM of TOP1MT, 40 nM of TOP3A, or 0.125, 0.25 or 0.5 U *E. coli* TopoI (New England Biolabs). All reactions were assembled on ice, initiated by the addition of ribonucleotides and incubated at 32°C. At the indicated times, 25 μl was removed from the master mix and reactions were ended by the addition of stop buffer (10 mM Tris–HCl pH 8, 200 mM NaCl, 1 mM EDTA and 100 μg/ml proteinase K) followed by incubation at 42°C for 45 min. Reaction products were purified by ethanol precipitation and pellets were resuspended in loading buffer (98% (v/v) formamide, 10 mM EDTA, 0.025% (w/v) xylene cyanol and 0.025% (w/v) bromophenol blue). Transcripts from supercoiled templates were resolved on denaturing 4% (v/v) polyacrylamide gels, and RNAs synthesised from a linearised dual promoter substrate were resolved on 10% (v/v) polyacrylamide gels. Gels were exposed to phosphorimager screens and imaged using a FLA-7000 scanner.

### DNA relaxation assays

Relaxation experiments (20 μl) contained 25 mM Tris–HCl (pH 8.0), 10 mM MgCl_2_, 100 μg/ml BSA, 1 mM DTT and 5 nM of supercoiled DNA template. Before the addition of the topoisomerase, DNA substrates were preincubated at 37°C for 10 min in the absence or presence of mtSSB as indicated in the figures (6.25, 12.5, 25, 50, 100 or 200 nM for titration experiments and 200 nM for time-course assays. Similarly, when TFAM was assayed, DNA was preincubated with 250 nM TFAM alone or together with mtSSB (125, 250 or 500 nM), then 25 nM (or 50 nM for the TFAM containing experiment) of TOP1MT was added to the reactions and incubated for 10 min. Reactions were terminated by the addition of stop buffer (10 mM Tris–HCl (pH 8), 1 mM EDTA, and 100 μg/ml proteinase K) and incubated at 42°C for 30 min. DNA SDS loading buffer (Thermo Scientific) was used to resolve the resulting topoisomers in 1× TAE, 0.8% (w/v) agarose gels. Gels were stained with 500 ng/ml ethidium bromide in TAE buffer and visualised in a UV transilluminator.

## RESULTS

### Two topoisomerases localise to the mitochondrial matrix in human cells

The reliable assignment of mitochondrial localisation to a protein relies upon a combination of multiple complementary techniques, which can include bioinformatic methods (the identification of a mitochondrial targeting sequence and evidence from the databases MitoCarta 3.0 (5) and/or MitoMiner (70)), and experimental methods (demonstration of localisation using cell fractionation and immunofluorescence, and a demonstrated functional effect upon mitochondria and mtDNA). The mitochondrial localisation of TOP1MT and TOP3A is supported by MitoCarta 2.0 and MitoMiner, and both bear N-terminal sequences with a high probability of mitochondrial localisation using different prediction servers (Supplementary Figure S1A). The remaining four topoisomerases are absent from MitoCarta 2.0, mitochondrial localisation is not supported by MitoMiner, and the presence of N-terminal targeting sequences could not be detected (Supplementary Figure S1A).

**Figure 1.**
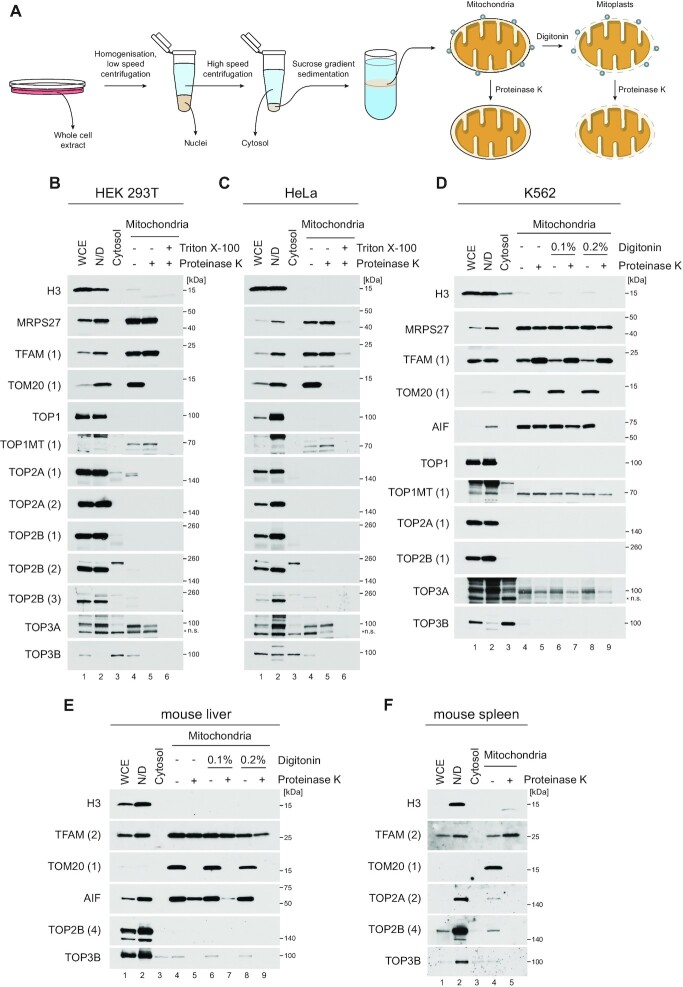
Subcellular localisation of human topoisomerases using cell fractionation. (**A**) Outline of method for mitochondrial isolation using differential centrifugation, and treatment of mitochondrial fractions with digitonin and proteinase K. (B, C) HEK293T cells (**B**) or HeLa cells (**C**) were fractionated using differential centrifugation to generate whole cell extract (WCE, lane 1), nucleus/debris (N/D, lane 2), cytosol (lane 3), and sucrose-gradient purified mitochondrial fractions (lane 4). Isolated mitochondria were further treated with proteinase K in the absence (lane 5) or presence (lane 6) of Triton X-100. Fractions were analysed using western blotting for each of the six human topoisomerases, as well as with markers for the nucleus (H3), mitochondrial matrix (MRPS27 and TFAM) and mitochondrial outer membrane (TOM20). *n.s. indicates non-specific bands. Where multiple antibodies to the same protein have been used, numbers in brackets indicate the identity of that antibody throughout the paper (see Supplementary Table S2). (**D**) K562 cells were fractionated and mitochondria isolated as in (B-C). Isolated mitochondria were then treated with proteinase K in the absence or presence of digitonin to selectively solubilise the outer mitochondrial membrane (lanes 4–9). Cellular fractions were blotted as in (B, C) with the addition of the mitochondrial intermembrane space marker protein AIF. (E, F) Localisation of topoisomerases from mouse tissues. Mouse liver (**E**) or spleen (**F**) were fractionated as in (B–D), and subcellular fractions were blotted with the indicated antibodies.

We experimentally studied the subcellular localisation of the six human topoisomerases by fractionating routinely used human cultured cell lines of varying origin. Mitochondria were purified by differential centrifugation and sucrose gradient centrifugation, and a portion of the mitochondrial fraction was treated with proteinase K to remove externally-bound protein contaminants (Figure 1A). Cellular fractions were blotted using marker proteins specific for different cellular compartments. This analysis corroborated the mitochondrial localisation of the well-characterised mitochondrial topoisomerase TOP1MT (16), as well as the dual nuclear and mitochondrial localisation of TOP3A (25,28) and exclusive nuclear localisation of TOP1 (45) in HEK293T (Figure 1B), HeLa (Figure 1C), SH-SY5Y (Supplementary Figure S1B) and K562 (Supplementary Figure S1C). Neither TOP2A nor TOP2B were detectable in proteinase K-treated mitochondrial extracts of HeLa and HEK293T cells using multiple antibodies (Figure 1B, C). In SH-SY5Y and K562 cells, low levels of TOP2A and TOP2B were visible in mitochondrial fractions only prior to proteinase K treatment, mirroring the distribution of the nuclear marker H3 and suggesting that this represents nuclear contamination (Supplementary Figure S1B, C). Extended exposures of these blots confirmed that all detectable TOP2A and TOP2B signal in mitochondrial fractions is removed by proteinase K treatment (Supplementary Figure S1D). We observed TOP3B localisation to both the nucleus and the cytosol, as previously described (30,31).

We additionally performed submitochondrial fractionation using K562 cells, using a combination of digitonin to solubilise the OMM and proteinase K to remove protein contaminants. The resistance of TOP1MT and TOP3A to protease treatment in these samples corroborated their mitochondrial matrix localisation, and we again found no evidence of mitochondrial localisation for TOP2A, TOP2B, TOP1 or TOP3B (Figure 1D and Supplementary Figure S1E). The localisation of TOP2A, TOP2B and TOP3B was further confirmed by fractionation of mouse liver (Figure 1E), and mouse spleen (Figure 1F). We were unable to detect TOP2A in adult mouse liver (Supplementary Figure S1F), consistent with a previous report that TOP2A is not expressed in this tissue (38). All detectable TOP2A, TOP2B and TOP3B signal in mitochondrial fractions of these tissues was again removed by proteinase K treatment, indicating that it does not correspond to mitochondrial matrix-localised protein. TOP2A, TOP2B and TOP3B were not degraded under the same incubation conditions as used for proteinase K treatment but without the enzyme (Supplementary Figure S1G), confirming that the removal of these proteins from proteinase K-treated samples is not the result of thermal degradation. The specificity of all topoisomerase antibodies used was confirmed by using siRNA transfections to downregulate each of the six topoisomerases in HeLa cells (Supplementary Figure S1H, I).

### Cellular localisation of human topoisomerases using confocal microscopy

**Figure 2.**
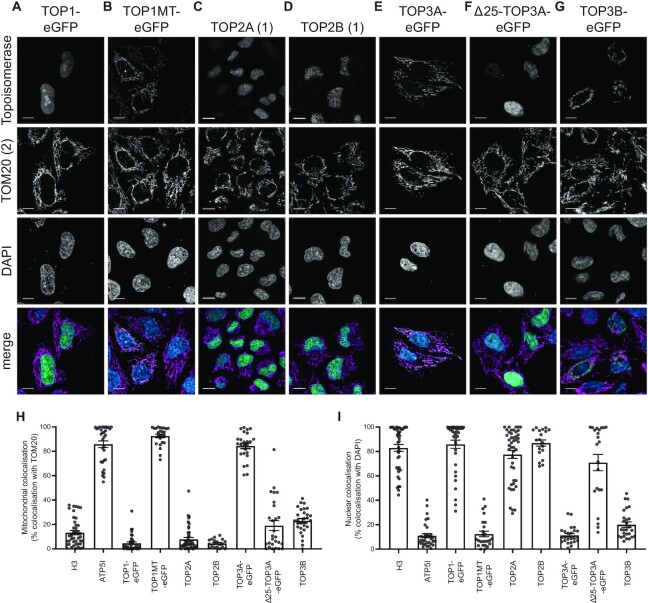
Subcellular localisation of human topoisomerases using Airyscan laser scanning microscopy. (A–G) Representative images showing the cellular localisation in cultured HeLa cells of (**A**) TOP1, (**B**) TOP1MT, (**C**) TOP2A, (**D**) TOP2B, (**E**) full-length TOP3A, (**F**) TOP3A lacking the N-terminal 25 amino acid mitochondrial targeting sequence (Δ25-TOP3A) and (**G**) TOP3B. TOP2A and TOP2B are imaged using antibodies specific to the endogenous proteins. All others are expressed as a fusion with C-terminal eGFP and imaged using GFP fluorescence. Images are shown as single-channel greyscale images for each topoisomerase (row 1), the mitochondrial marker TOM20 (row 2), DAPI (row 3), and a merge image (row 4, with the topoisomerase in green, TOM20 in magenta, and DAPI in blue). All scale bars represent 10 μm. (H, I) Quantification of the co-localisation of topoisomerase signal as in (A–G) with the mitochondrial marker TOM20 (**H**) or the nuclear marker DAPI (**I**). ATP5I is used as a positive control for mitochondrial co-localisation, and H3 is used as a positive control for nuclear co-localisation. Each data point represents one individual cell.

We next analysed the subcellular localisation of the six human topoisomerases using super-resolution Airyscan confocal microscopy. Suitable antibodies specific to endogenous TOP2A and TOP2B are available, and in both cases a nuclear, but not mitochondrial, localisation was detectable for both proteins in HeLa cells (Figure 2C, D) and in SH-SY5Y cells (Supplementary Figure S2A, B). The specificity of the TOP2B antibody used was confirmed using a SH-SY5Y TOP2B knockout cell line (Supplementary Figure S2C, D), in which a low level of non-specific background signal was still visible, indicating some cross-reactivity of the antibody. For TOP1, TOP1MT, TOP3A and TOP3B, proteins were transiently expressed with a C-terminal fusion of eGFP. TOP1 again showed an exclusively nuclear localisation (Figure 2A), and TOP1MT an exclusively mitochondrial localisation (Figure 2B). The *TOP3A* gene encodes both the nuclear and mitochondrial isoforms (25). We found that expression of the full-length cDNA of TOP3A fused to eGFP led to an exclusively mitochondrial localisation, whereas expression of a truncated form of TOP3A lacking the first 25 amino acids (aa, Δ25-TOP3A, representing the nuclear isoform) led to an exclusively nuclear localisation (Figure 2E, F). TOP3B-eGFP showed a predominantly cytosolic localisation pattern that did not colocalise with the mitochondrial network (Figure 2G), possibly representing co-localisation with cytosolic translation. Quantification of the co-localisation of topoisomerase signal with the nucleus (using DAPI) or mitochondria (using TOM20) confirmed that TOP1MT and TOP3A show a comparable localisation pattern to the mitochondrial marker ATP5I (Figure 2H and Supplementary Figure S3A), and TOP1, TOP2A, TOP2B and Δ25-TOP3A show a similar localisation to the nuclear marker H3 (Figure 2I and Supplementary Figure S3B). Cells incubated with secondary antibodies only (Supplementary Figure S3C, D) and controls for autofluorescence (Supplementary Figure S3E) were used to confirm the specificity of the observed signal.

### TOP3A and TOP1MT both contribute to mtDNA replication

**Figure 3.**
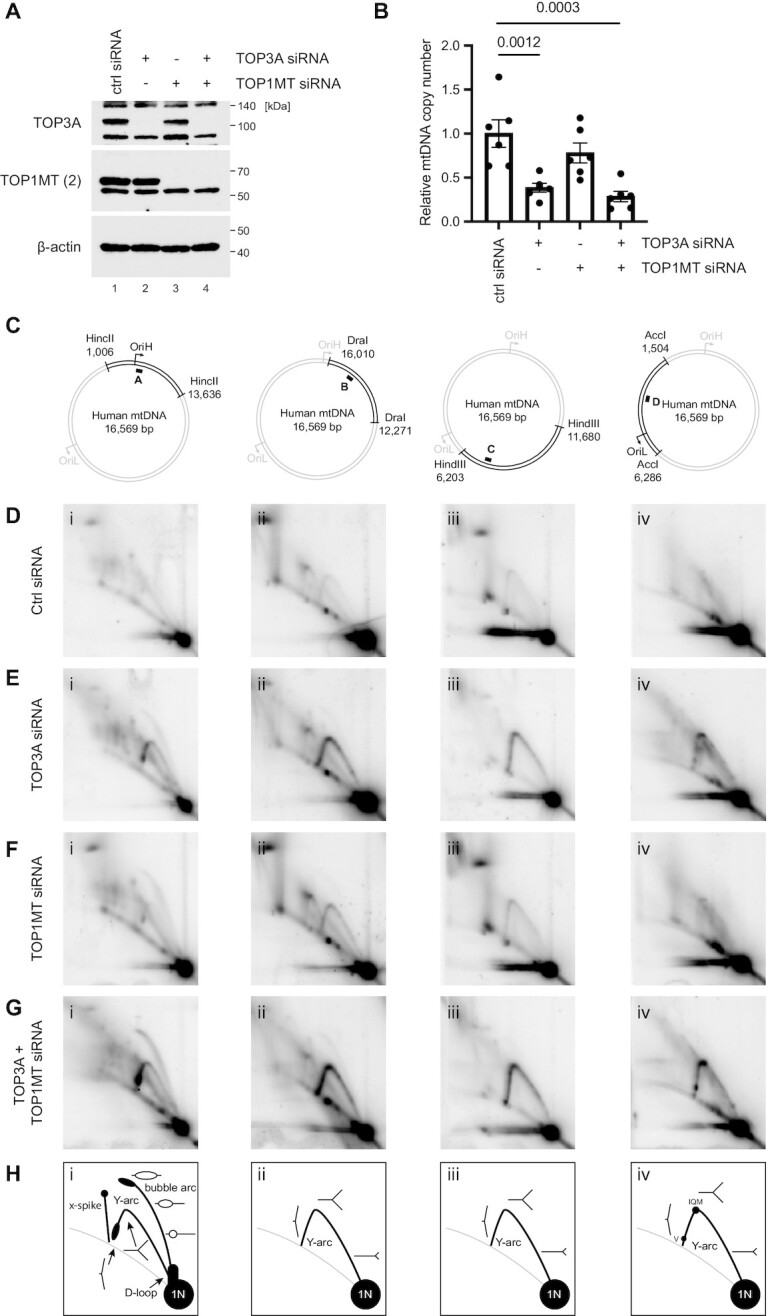
Loss of TOP3A or TOP1MT impairs mtDNA replication progression. (**A**) Western blot of HeLa cell lysates following siRNA depletion of TOP3A and TOP1MT, either alone or in combination. β-actin is used as a loading control. (**B**) mtDNA copy number in TOP3A and TOP1MT-depleted cells as in (A) measured using qPCR. Copy number is expressed as the level of an mtDNA-specific amplicon (ND1) normalised to the level of a nuclear amplicon (B2M) normalised to the control siRNA sample. Plot shows mean values, *n* = 6, error bars represent ± SEM, p values are given from one-way ANOVA. (**C-H**) Mitochondrial DNA replication patterns in topoisomerase-depleted analysed using 2DNAGE. (**C**) mtDNA was restricted using the indicated enzymes. The black bar indicates the location of the probe. (D–G) 2DNAGE blots of mtDNA in cells treated with control siRNA (**D**), TOP3A siRNA (**E**), TOP1MT siRNA (**F**) or TOP1MT + TOP3A siRNA (**G**). (**H**) Diagrams indicating prominent replication intermediates observed using 2DNAGE.

We next investigated the contributions of the mitochondrial topoisomerases TOP3A and TOP1MT to mtDNA replication. As TOP3A is an essential gene in mammals (71), we used siRNA to deplete the levels of TOP3A and TOP1MT, either separately or simultaneously, in HeLa cells (Figure 3A). The loss of TOP3A, but not TOP1MT, caused a depletion of mtDNA copy number (Figure 3B) as observed previously (28). We then used 2D neutral agarose gel electrophoresis (2DNAGE) to monitor mtDNA replication patterns, using four restriction fragments to cover almost the entire mtDNA (Figure 3C). The loss of either TOP3A (Figure 3E), and to a lesser degree TOP1MT (Figure 3F), caused an accumulation of mtDNA replication intermediates throughout the mitochondrial genome. Given that mtDNA copy number is either unchanged or reduced in these cells (Figure 3B), this accumulation of mtDNA replication intermediates can be interpreted as a non-site-specific replication stalling phenotype, indicative of impaired mtDNA replication fork progression. This replication stalling phenotype was exacerbated in cells depleted of both TOP3A and TOP1MT simultaneously (Figure 3G), supporting the notion that both topoisomerases contribute to mtDNA replication. Interestingly, bubble arcs (indicative of replication initiation) were visible in topoisomerase-depleted cells in a DraI restriction fragment that lacks the canonical replication origin OriH (Supplementary Figure S4C–E). Replication initiation in this region has been previously observed at low levels in solid tissues (72), and suggests that changes to mtDNA topology results in alterations to the location of mtDNA replication initiation. In addition to this generalised inhibition of replication progression, prominent sites of specific mtDNA replication pausing or stalling were also visible in the AccI digest in topoisomerase-depleted cells, corresponding approximately to the IQM tRNA cluster in the mtDNA minor arc, as well as the region corresponding to mt-tRNA^Val^ (Figure 3E and G, panel iv). A large accumulation of replication intermediates is also visible on the descending portion of the y-arc in the HincII digest in TOP3A and TOP3A/TOP1MT-depleted cells, corresponding to late-stage replication intermediates in the region of OriH (Figure 3E, panel i, and G, panel i).

**Figure 4.**
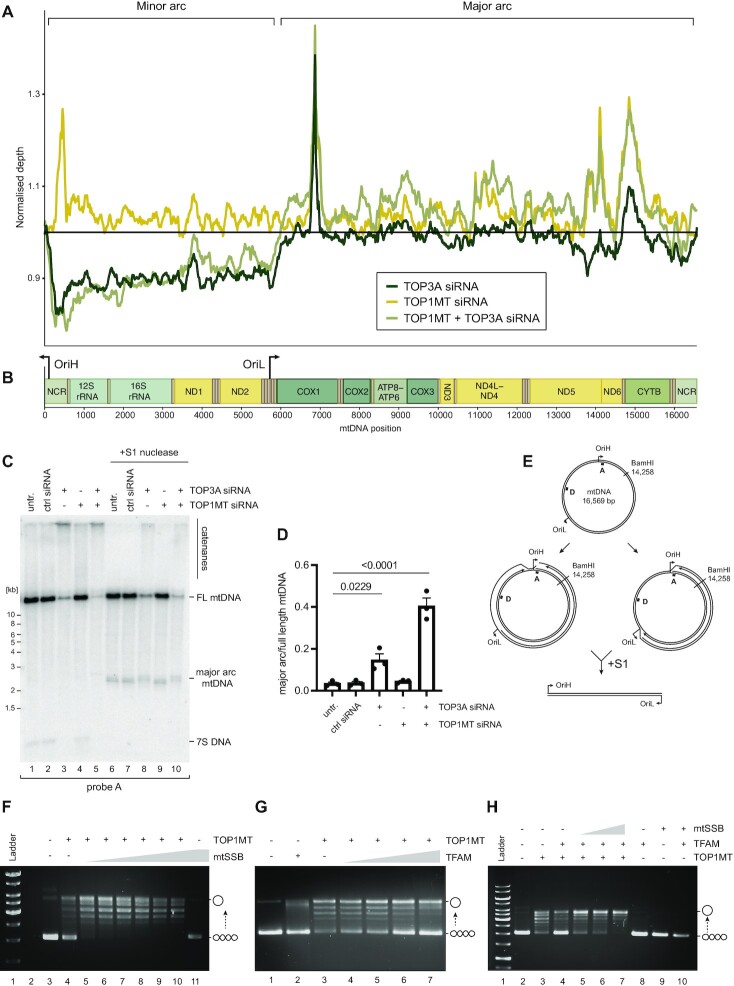
Topoisomerase-associated mtDNA replication phenotypes. (**A**) Normalised mtDNA whole genome sequencing coverage from topoisomerase-depleted HeLa cells. (**B**) Schematic of mtDNA indicating gene loci and mtDNA replication origins. (**C**) Southern blot of full-length and major arc mtDNA from topoisomerase-depleted cells. Total DNA was linearised with BamHI and then left untreated (lanes 1–5) or treated with S1 nuclease (lanes 6–10), then Southern blotted using probe A, which detects full-length mtDNA, the released major arc fragment, and 7S DNA. (**D**) Quantification of the proportion of major arc mtDNA produced by S1 nuclease treatment as in (C), normalised to the level of full-length mtDNA. Plot shows mean values, n = 3, error bars represent ± SEM, p values are given from one-way ANOVA. (**E**) Graphical representation of mtDNA structures potentially giving rise to phenotypes as in (C, D). (F–H) Effects of mtSSB and TFAM upon TOP1MT activity *in vitro*. (**F**) Negatively supercoiled pUC18 DNA was incubated with 25 nM recombinant TOP1MT either in the absence (lane 4) or the presence (lanes 5–10) of increasing concentrations of mtSSB (6.25, 12.5, 25, 50, 100 and 200 nM), separated by agarose gel electrophoresis, and stained using ethidium bromide. The migration of supercoiled and relaxed substrate DNA is indicated. (**G**) pUC18 DNA as in (F) was incubated with 50 nM recombinant TOP1MT in the absence (lane 3) or presence (lanes 4–7) of increasing concentrations of TFAM (5, 10, 25 and 50 nM). (**H**) pUC18 DNA as in (F) was incubated with 50 nM recombinant TOP1MT and 250 nM TFAM in the presence of different concentrations of mtSSB (125, 250 and 500 nM) as indicated.

We further analysed the contribution of TOP3A and TOP1MT to mtDNA replication using whole genome sequencing of DNA from topoisomerase-depleted cells. The sequence coverage patterns in topoisomerase-depleted cells were plotted normalised to control cells, which indicated that cells depleted of TOP3A (either alone or in combination with TOP1MT) showed a reduction in the level of minor arc mtDNA relative to major arc mtDNA (Figure 4A, B and Supplementary Figure S5A). The presence of free, linear major arc mtDNA has previously been associated with the loss of POLγ proofreading activity in mice (73) and in patients and mice lacking the mitochondrial exonuclease MGME1 (74,75). However, Southern blotting of DNA from topoisomerase-depleted cells did not show any evidence of linear major arc mtDNA (Figure 4C, lanes 1–5). Conversely, high proportions of linear mtDNA could be released upon S1 nuclease treatment of DNA from TOP3A depleted cells, visible when probing for the mtDNA major arc (Figure 4C, lanes 6–10) but not the minor arc (Supplementary Figure S5B) (76), indicating that the increased proportion of major arc mtDNA in TOP3A-depleted cells represents the accumulation of stalled but intact mtDNA replication intermediates (Figure 4E).

To further characterise the effect of TOP1MT upon mtDNA replication, we isolated recombinant TOP1MT and assessed its activity in the presence of key mtDNA-interacting proteins *in vitro*. TOP1MT was able to relax a negatively supercoiled pUC18 plasmid, with the activity being stimulated by the addition of the abundant and core mtDNA replication protein mtSSB (Figure 4F and Supplementary Figure S5C). Interestingly, the mtDNA packaging factor TFAM suppressed the relaxation activity of TOP1MT (Figure 4G), although this inhibition could be overcome by the addition of mtSSB (Figure 4H). These results suggest that the relaxation activity of TOP1MT may be directed preferentially towards replicating rather than packaged mtDNA.

### TOP3A and TOP1MT contribute to mitochondrial transcription

**Figure 5.**
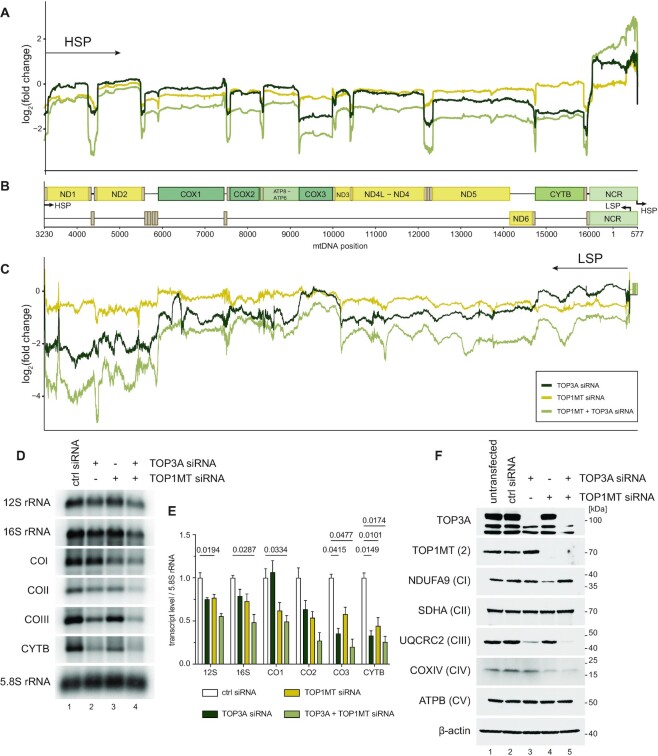
Loss of mitochondrial topoisomerases impairs mitochondrial gene expression. (A–C) RNA-seq coverage of mitochondrial transcripts following depletion of TOP3A and TOP1MT, alone or in combination, in HeLa cells. Depth profiles from HSP-derived transcripts (**A**) and LSP-derived transcripts (**C**) are shown normalised to control siRNA-treated cells. Gene and promoter loci are indicated in (**B**) Note that mt-rRNAs are depleted by the library preparation and so are omitted. (**D**) Representative northern blots of mitochondrial transcript levels in TOP3A and TOP1MT depleted cells as in (A-C). 5.8S rRNA is used as a loading control. (**E**) Quantification of mitochondrial RNA levels from northern blots as in (D). Data represent mean ± SEM, *n* = 3, p values are given from one-way ANOVA. (**F**) Levels of respiratory complex proteins following depletion of TOP3A and TOP1MT assessed using western blotting. One marker protein is used for each of complexes I–V (CI-CV), and β-actin is used as a loading control.

We next considered the contributions of TOP3A and TOP1MT towards mitochondrial gene expression. We first determined the effects of loss of topoisomerase activity upon the steady-state levels of individual mitochondrial RNAs using RNA-seq (Figure 5A–C and Supplementary Figure S6A, B). The counts of TOP3A and TOP1MT RNAs corroborated the loss of these transcripts in siRNA-treated cells (Supplementary Figure S6C, D). The steady-state levels of mtDNA transcripts derived from HSP appeared to be reduced in TOP3A and TOP3A/TOP1MT depleted cells, and showed a particularly large decrease in transcripts distal to the promoter (Figure 5A). The levels of LSP-derived promoter-distal RNAs, mostly representing non-coding antisense RNAs, were also decreased in TOP3A and TOP3A/TOP1MT siRNA-treated cells (Figure 5C). We confirmed the loss of transcript levels, particularly promoter-distal transcripts, using northern blotting (Figure 5D, E), which also showed a moderate loss of transcript levels in TOP1MT-depleted cells. Differentially expressed genes are provided in Supplementary Table S4. An analysis of the levels of respiratory chain complex subunits in topoisomerase-depleted cells (Figure 5F) found a profound loss of respiratory complex III in the absence of TOP3A, possibly reflecting the loss of the most HSP-distal *Cytb* transcript. Taken together, these data indicate that mitochondrial transcription is impaired in the absence of either TOP3A or TOP1MT, with the loss of TOP3A also resulting in impaired processivity.

**Figure 6.**
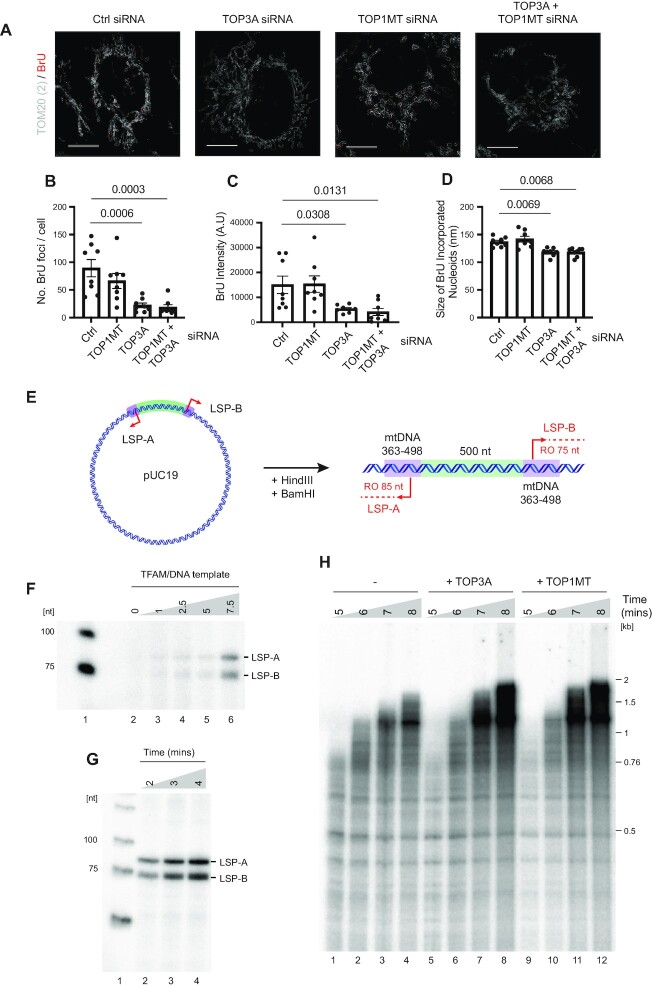
TOP3A and TOP1MT contribute to mitochondrial transcription progression. (**A**) Representative STED microscopy images showing co-staining of BrU incorporation (red) and mitochondria (TOM20, white) following the indicated siRNA treatments. (B–D) Quantifications of the number of BrU foci per cell (**B**), fluorescence intensity of BrU foci (**C**) and size of BrU foci (**D**) as in (A). Data represent mean measurements ± SEM, n = 8, p values from one-way ANOVA are indicated. (E–H) Construction and validation of a dual promoter template for *in vitro* mitochondrial transcription. (**E**) Diagram of the linearised template showing the expected size of runoff *in vitro* transcription products from LSP-A and LSP-B. (**F**) *In vitro* transcription reactions using the template as in (E), showing relative proportions of LSP-A and LSP-B transcription products. Reactions contained POLRMT, TFB2M, and increasing concentrations of TFAM as indicated (TFAM/DNA ratios of 0, 1, 2.5, 5 and 7.5, corresponding to 0, 4, 10, 20 and 30 nM TFAM respectively). (**G**) Time course of runoff *in vitro* transcription reactions using the dual promoter template as in (E). (**H**) *In vitro* transcription reactions using the supercoiled template as in (E). Reactions contained POLRMT, TFAM, TFB2M and TEFM in the absence (lanes 1–4) or presence of 40 nM TOP3A (lanes 5–8) or 10 nM TOP1MT (lanes 9–12) as indicated.

We next analysed the effects of topoisomerase depletion upon *de novo* transcription activity by pulse-labelling cells with BrU and imaging using STED super-resolution microscopy (Figure 6A). The loss of TOP3A, either alone or in combination with TOP1MT, caused a significant loss of the number of BrU foci per cell, consistent with the loss of mtDNA copy number per cell (Figure 6B) but also a loss of BrU intensity (Figure 6C) and foci size (Figure 6D), indicative of impaired overall transcription rate.

It is possible that this effect of TOP3A upon mitochondrial transcription reflects a role for TOP3A in transcription itself. However, given the role of TOP3A in the decatenation of mtDNA replication products (28), it may be that the impaired transcription processivity in TOP3A-depleted cells results from a difficulty in transcribing catenated mtDNA molecules. To distinguish these possibilities, we used a reconstituted *in vitro* mitochondrial transcription system consisting of the mitochondrial RNA polymerase POLRMT, two essential transcription factors TFAM and TFB2M (67), and transcription elongation factor TEFM (68). We constructed plasmid substrates containing two copies of LSP in opposing orientations in order to create two converging transcription units with domains of negative and positive supercoiling (Figure 6E). The two copies of LSP (LSP-A and LSP-B) were found to be activated by equal concentrations of TFAM (Figure 6F), and synthesised equimolar runoff transcription products in time-course experiments (Figure 6G and Supplementary Figure S6E) using linearised plasmid template. As a control, the addition of the *E. coli* type IA topoisomerase TopoI stimulated the formation of transcription products using this *in vitro* system (Supplementary Figure S6F). When either TOP3A or TOP1MT were added to these reactions, a modest increase in both the amount and length of transcription products was observed (Figure 6H), supporting the notion that both TOP3A and TOP1MT can catalyse alterations to DNA topology during mitochondrial transcription.

### TOP2B does not directly contribute to mtDNA maintenance or expression

**Figure 7.**
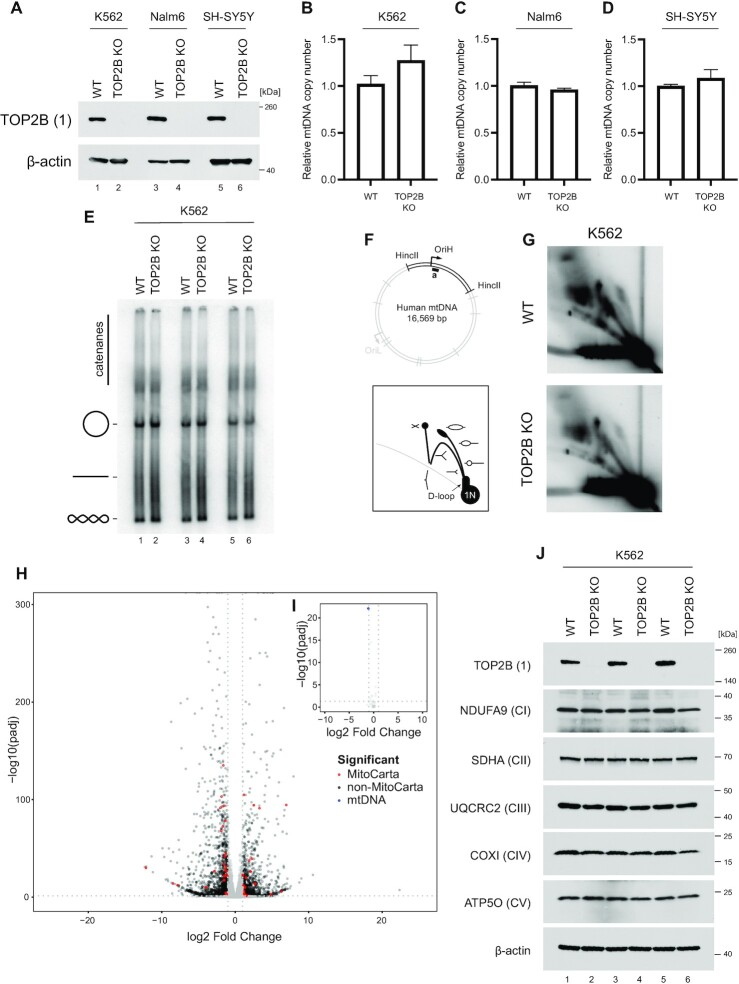
TOP2B does not directly contribute to mtDNA maintenance. (**A**) TOP2B knockout lines of Nalm6, K562, and SH-SY5Y were created using CRISPR-Cas9. TOP2B levels were assessed using western blotting; β-actin is used as a loading control. (B–D) mtDNA copy number in K562 (**B**), Nalm6 (**C**) and SH-SY5Y (**D**) WT and TOP2B knockout cells assessed using qPCR. The amplification of mtDNA (ND1) is normalised to the level of nuclear DNA (B2M) and normalised to the wild-type control. Bars represent mean values, *n* = 3, error bars represent ± SEM expressed relative to ratios. (**E**) mtDNA topology following knockout of TOP2B in K562 cells. Uncut mtDNA was separated on agarose gels and Southern blotted using an mtDNA-specific probe. The migration of open-circle form, linear and supercoiled mtDNA is indicated. (**F–G**) Assessment of mtDNA replication patterns in WT and TOP2B knockout K562 cells assessed using 2DNAGE. The diagram indicates the location of the analysed HincII restriction fragment and probe (top panel) and schematic of observed mtDNA replication intermediates (bottom panel). (**H**) Volcano plot showing expression of nuclear-encoded genes in SH-SY5Y TOP2B knockout cells relative to a WT control. Differentially expressed genes (log_2_FC > 1, –log_10_(*P*_adj_) > 0.05) are coloured red or black according to their presence or absence (respectively) in MitoCarta. (**I**) Volcano plot showing the expression of mtDNA-encoded genes in SH-SY5Y TOP2B knockout cells as in (H). Differentially-expressed genes are coloured in blue. (**J**) Levels of respiratory chain complex proteins following knockout of TOP2B in K562 cells assessed using western blotting. One marker protein is used for each of complexes I–V, and β-actin is used as a loading control. Replicates represent separate protein extractions from sequential passages of each cell line.

In order to further study the potential functional effects of TOP2 activity upon mtDNA, we generated TOP2B knockout cell lines of K562, Nalm6 and SH-SY5Y using CRISPR/Cas9 (Figure 7A). We have concentrated upon TOP2B because of its ubiquitous expression and because of its previously proposed role in mtDNA metabolism (44–46). As TOP2A is only expressed in dividing cells it would be unable to make an essential contribution to mtDNA replication, which takes place throughout the cell cycle as well as in post-mitotic cells (77). An analysis of mtDNA copy number using qPCR found no differences between wild-type cells and TOP2B knockout cells (Figure 7B–D). We next analysed mtDNA topology following knockout of TOP2B. Loss of mitochondrial topoisomerase activity may manifest as either an alteration of mtDNA supercoiling or as an impairment of mtDNA decatenation (visualised as a loss of monomeric mtDNA forms), both of which can be observed by blotting uncut mtDNA. This analysis found no alterations to mtDNA structure or topology upon TOP2B knockout (Figure 7E and Supplementary Figure S7A, B). We then analysed the pattern of mtDNA replication intermediates using 2DNAGE (Figure 7F, G and Supplementary Figure S7C–E). No clear differences were observed between wild-type and TOP2B knockout cells, indicating that TOP2B is not necessary for mtDNA replication progression. In order to study the effects of the loss of TOP2B upon mitochondrial gene expression, we analysed RNA-seq data from these WT and TOP2B knockout SH-SY5Y cells (78), and filtered nuclear-encoded differentially expressed genes according to the mitochondrial protein database MitoCarta (Figure 7H, I and Supplementary Table S5). This analysis found a number of nuclear-encoded mitochondrial proteins to be differentially expressed (Figure 7H), but not mtDNA-encoded proteins themselves (Figure 7I), suggesting that alterations to mitochondrial metabolism in these cells are mediated through changes to nuclear gene expression.

We analysed the levels of respiratory chain complex proteins in TOP2B knockout cells using western blotting, which did not find any changes in the levels of marker proteins for each of the five complexes compared with wild-type cells (Figure 7J and Supplementary Figure S7F, G). It has previously been suggested that TOP2A could compensate for the absence of TOP2B within mitochondria (46). In order to account for this possibility, we depleted cells of TOP2A and TOP2B, either individually or simultaneously. We found no changes to either mtDNA copy number (Supplementary Figure S7H) or the expression of OXPHOS proteins (Supplementary Figure S7I), indicating that there is no adaptive response in mitochondria involving TOP2A in cells lacking TOP2B, consistent with our subcellular localisation data that finds no evidence for mitochondrial localisation of these proteins.

## DISCUSSION

Our results indicate that the maintenance of human mtDNA topology relies on only two topoisomerases, TOP1MT and TOP3A, and that both enzymes contribute to mtDNA replication and transcription. TOP1MT, as a type IB topoisomerase, is capable of relieving either positive supercoils (overwinding of DNA) or negative supercoils (underwinding of DNA) that arise during transcription and DNA replication (16,17). TOP3A, in contrast, is only capable of removing negative supercoils because of the requirement of type IA topoisomerases to act upon single-stranded regions of DNA (79). In this regard it is interesting to note that TOP1MT is not an essential gene, with knockout mice showing no obvious physical phenotypes despite mtDNA supercoiling being altered (45,80). This may indicate that, despite TOP1MT playing a regulatory role during transcription, the accumulation of positive supercoiling does not pose a significant problem for mtDNA. TOP3A, as a type IA topoisomerase, is also involved in the processing of nuclear recombination intermediates as part of the dissolvasome (81). Although mitochondria appear to lack a mechanism for homologous recombination (82), it is possible that TOP3A activity could also help to supress the formation of such intermediates, which may be toxic within mitochondria.

**Figure 8.**
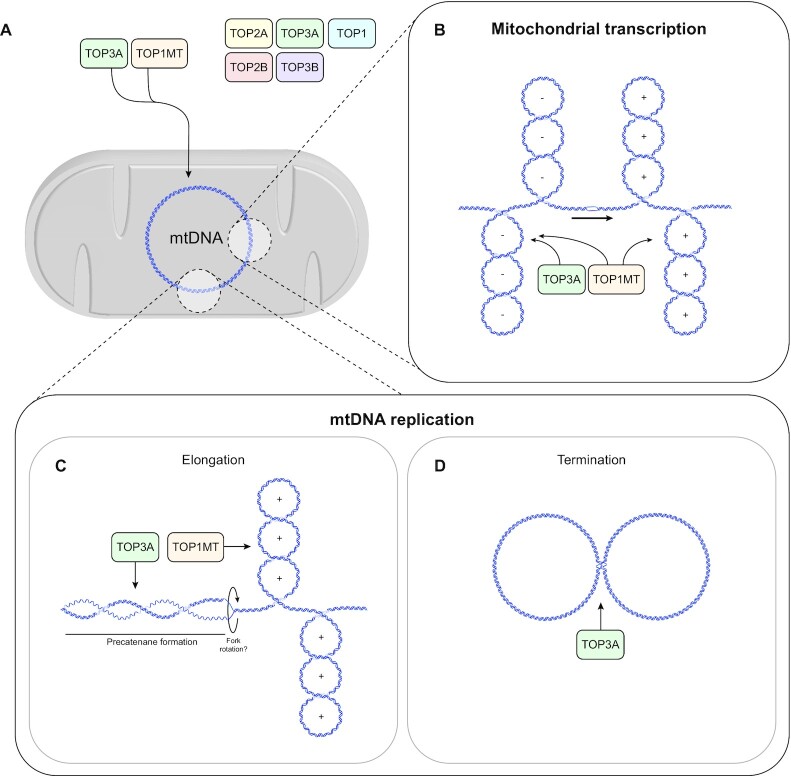
Model for topoisomerase roles in mtDNA topology maintenance. (**A**) TOP1MT and TOP3A localise to the mitochondrial matrix in human cells. (**B**) TOP3A facilitates processive mitochondrial transcription, presumably through the removal of negative supercoiling, together with TOP1MT. (**C**) TOP3A and TOP1MT removing supercoiling during mtDNA replication, which promotes replication fork progression. (**D**) TOP3A is required for the decatenation of mtDNA replication products.

Our results, together with the known substrate specificities of TOP3A and TOP1MT, suggest a division of labour between the two proteins during mitochondrial transcription and DNA replication (Figure 8).

During transcription, the DNA template ahead of the transcription machinery becomes positively supercoiled, while the DNA behind becomes negatively supercoiled (10). Problems associated with transcription-associated supercoiling are exacerbated in situations in which superhelical tension cannot be relieved by the rotation of the DNA molecule around its helical axis (10). This includes the presence of transcription units oriented in opposing directions, or by the existence of topologically-restricted domains created by attachment to a membrane. Human mtDNA promoters are responsible for polycistronic transcription of almost the whole genome in both directions (1,83). However, it remains unclear whether promoters are active simultaneously, as individual regulation of promoter firing could mitigate the effects of topological stress during transcription. It is also unclear to what degree and in what location mtDNA associates with the inner mitochondrial membrane (29,84). Although mtDNA nucleoids can be found tightly apposed to the membrane (85), nucleoids may only associate transiently with the membrane, for example during DNA replication (86), which again may mitigate the effects of supercoiling. TOP1MT has an established role in regulating mitochondrial transcription (17–19). Interestingly, some previous studies have found elevated levels of mtDNA transcripts in the absence of TOP1MT (17,19), whereas we found that mitochondrial transcripts are unaffected or depleted (Figure 5), suggesting a role more analogous to that of nuclear TOP1. Some of these effects may be accounted for by differences between the short-term effects seen in siRNA experiments and long-term adaptive responses in knockout models. Our results additionally suggest that TOP3A can contribute to transcription. Cells lacking mitochondrial TOP3A activity show a loss of transcription processivity which, because mitochondrial transcription is polycistronic, results in a selective depletion of promoter-distal transcripts (Figure 5). Mitochondrial DNA is catenated in the absence of TOP3A, which may contribute to the observed transcription defects (28). However, we also find that TOP3A and TOP1MT are both able to promote transcription elongation using an *in vitro* model of converging mitochondrial transcription units (Figure 6), suggesting that this activity may also be relevant *in vivo*. A deeper understanding of the regulation of mitochondrial transcription will therefore aid our understanding of mtDNA topology.

Topoisomerases also play crucial roles during DNA replication. The unwinding of template DNA by a helicase creates positive supercoiling ahead of the replication fork. This supercoiling may be relieved by rotation of the replisome, forming intertwined daughter DNA molecules behind the replisome that are referred to as precatenanes. During strand-coupled DNA replication of circular genomes, these linkages are composed of dsDNA and will be converted into catenanes if left unresolved at replication termination (87). A type II topoisomerase activity is required for decatenation of these replicated DNA molecules, because a double-strand break is required to remove either precatenanes behind the replication fork or fully catenated molecules following the completion of replication (87,88). However, type IA topoisomerases are also capable of acting as replicative decatenases provided that single-stranded gaps are present in the template for the topoisomerase to act upon (24,89–91). mtDNA is replicated by an unusual asynchronous mechanism whereby unidirectional leading-strand synthesis must pass the origin of lagging-strand replication before DNA synthesis can be initiated from this site (1,2). This creates a substantial delay between the initiation of leading- and lagging-strand synthesis from distinct sites on the genome. It is conceivable that this mechanism would generate the ssDNA gaps required for TOP3A to act as the sole decatenase in mitochondria (26,47). Such a model would be consistent with our results indicating that the loss of TOP3A results in the accumulation of hemicatenated replication termination intermediates (28). Our results indicate that the loss of either TOP3A or TOP1MT impairs mtDNA replication progression, with synergistic defects occurring in the absence of both proteins. As well as a genome-wide accumulation of mtDNA replication intermediates, site-specific stalling is also seen at tRNA sites in the mtDNA minor arc. TOP1MT could contribute to mtDNA replication through the removal of either positive or negative supercoiling during mtDNA replication progression. The observation that the DNA relaxation activity of TOP1MT is stimulated by the core mtDNA replication protein mtSSB *in vitro* (Figure 4) suggests the possibility that the activity of TOP1MT could be specifically directed towards replicating mtDNA. Interestingly, the loss of TOP3A was also associated with the presence of mtDNA replication initiation downstream of the canonical replication origin OriH (Supplementary Figure S4). Similar initiation events have previously been observed in solid tissues (72), and may indicate that alternate sites of mtDNA replication initiation are utilised in some circumstances. The over-replication of the mtDNA major arc has also previously been observed in the mutator mouse (73) and in patients with loss-of-function mutations in the mitochondrial nuclease MGME1 (74). However, in these other cases the major arc mtDNA was released from the genome, which is proposed to result from the presence of persistent unresolved nicks at the replication origins OriH and OriL (92). The latter stages of mtDNA replication, particularly the replication of the minor arc, remain poorly understood and may involve additional regulatory steps that are yet to be characterised.

Collisions between the transcription and replication machineries also present potential topological problems for mtDNA. Although the relative regulation of transcription and DNA replication in mitochondria remain poorly understood, recent data has indicated that both processes can occur simultaneously in individual nucleoids (93), and represents an interesting topic for further study.

We did not find any evidence for mitochondrial localisation of TOP2A or TOP2B in our experiments, which contrasts with previous reports of the localisation of TOP2 isoforms to mitochondria based either on activity (40–43) or through experimental visualisation (44–46). We find that protease treatment of isolated mitochondria is essential to remove traces of nuclear and cytosolic proteins even from sucrose gradient-purified and/or digitonin-treated mitochondrial preparations. We suggest that such treatments should be incorporated into any standard protocol seeking to demonstrate a mitochondrial matrix localisation of a protein in cultured cells. We also did not observe any mtDNA phenotypes following genetic ablation of TOP2B (Figure 7), consistent with our proposed model that TOP3A and TOP1MT are together capable of fulfilling all required topological transactions of human mitochondrial DNA.

## DATA AVAILABILITY

RNA-seq data from TOP3A and TOP1MT siRNA-treated cells has been deposited into the Gene Expression Omnibus (GEO) under accession number GSE201426. RNA-seq data from TOP2B knockout cells is available under accession number GSE142383. Whole genome sequencing data has been deposited into the Sequence Read Archive (SRA) under project number PRJNA831603. Raw image data associated with this paper is available at Zenodo and can be accessed using the DOI: 10.5281/zenodo.7115828.

## Supplementary Material

gkac857_Supplemental_Files

## Notes

Present address: Mushtaq M. Khazeem, National Center of Hematology, Mustansiriyah University, Baghdad, Iraq.
