# Supplementary material for: Two type I topoisomerases maintain DNA topology in human mitochondria
Source: Nucleic Acids Res. 2022 Oct 10;50(19):11154–74. doi: 10.1093/nar/gkac857 (PMC9638942; doi:10.1093/nar/gkac857)
Supplement: gkac857_Supplemental_Files [file gkac857_supplemental_files.zip › 020922_Menger_NAR_Revision_Supplementary_Information.pdf]

Supplementary Materials for

Two type I topoisomerases maintain DNA topology in human mitochondria

Katja E. Menger, James Chapman, Héctor Díaz-Maldonado, Mushtaq M. Khazeem, Dasha Deen, Direnis Erdinc, John W. Casement, Valeria Di Leo, Angela Pyle, Alejandro Rodríguez-Luis, Ian Cowell, Maria Falkenberg, Caroline A. Austin, and Thomas J. Nicholls*

Corresponding author. Email: thomas.nicholls@newcastle.ac.uk

Figure S1. Additional localisation of human topoisomerases using computational methods and cell fractionation.
Fig. S2. Further localisation of TOP2 isoforms using confocal microscopy.
Fig. S3. Controls for localisation experiments using microscopy.
Fig. S4. Extended exposures of 2DNAGE blots.
Fig. S5. Roles of TOP3A and TOP1MT in mtDNA replication.
Fig. S6. Roles of TOP3A and TOP1MT in mitochondrial transcription.
Fig. S7. Characterisation of TOP2B-knockout and TOP2A/TOP2B-depleted cells.
Table S1. siRNA oligonucleotides used in this study.
Table S2. Antibodies used in this study.
Table S3. Oligonucleotides for qPCR and Southern blotting.
Table S4. Differentially expressed genes in topoisomerase depleted cells.
Table S5. Differentially expressed genes in TOP2B knockout SH-SY5Y cells.

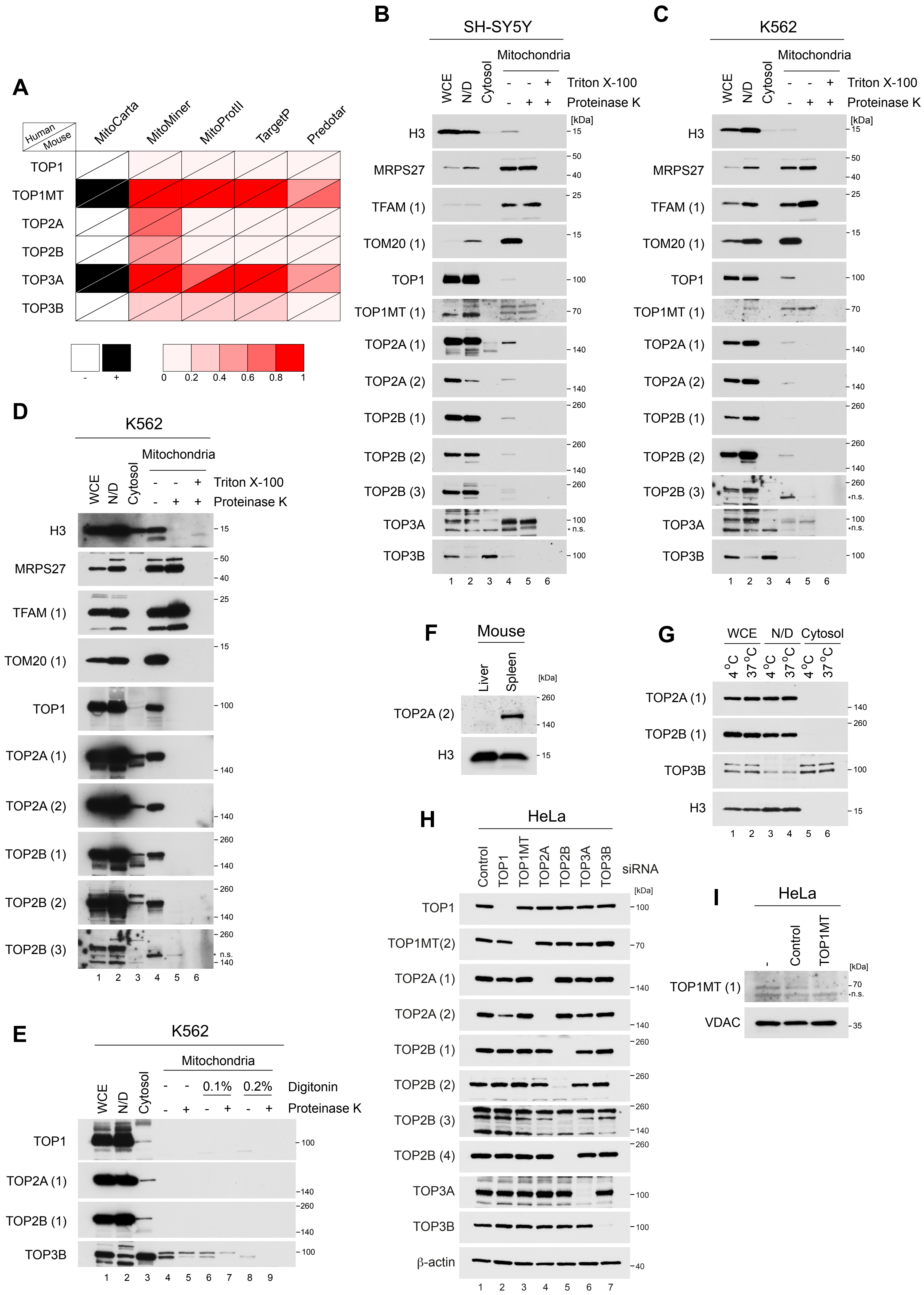
A
Human
Mouse
MitoCarta
MitoMiner
MitoProtII
TargetP
Predotar
TOP1
TOP1MT
TOP2A
TOP2B
TOP3A
TOP3B
-
+
0
0.2
0.4
0.6
0.8
1
B
SH-SY5Y
C
K562
WCE
N/D
Cytosol
Mitochondria
Triton X-100
Proteinase K
[kDa]
H3
MRPS27
TFAM (1)
TOM20 (1)
TOP1
TOP1MT (1)
TOP2A (1)
TOP2A (2)
TOP2B (1)
TOP2B (2)
TOP2B (3)
TOP3A
TOP3B
n.s.
1 2 3 4 5 6
D
K562
E
K562
Digitonin
0.1%
0.2%
1 2 3 4 5 6 7 8 9
F
Mouse
Liver
Spleen
G
4 °C
37 °C
H
HeLa
Control
siRNA
TOP1MT(2)
TOP2B (4)
β-actin
1 2 3 4 5 6 7
I
HeLa
VDAC

Figure S1. *Additional localisation of human topoisomerases using computational methods and cell fractionation.*

(A) Computational predictions of mitochondrial localisation for the six topoisomerases in humans and in mice. Triangles representing the human (top left) or mouse (bottom right) topoisomerases are coloured according to the presence (black) or absence (white) in MitoCarta 2.0 (column 1). Proteins are also colour-coded according to scores generated by the integrative mitochondrial protein database MitoMiner (column 2) and the N-terminal mitochondrial targeting sequence prediction servers MitoProtII, TargetP and Predotar (columns 3-5).

(B-C) Subcellular localisation of human topoisomerases using cell fractionation in SH-SY5Y cells **(B)** and K562 cells **(C)**. Cells were disrupted and fractionated using differential centrifugation as in Figure 1, generating fractions for whole cell extract (WCE, lane 1), nuclei/debris (N/D, lane 2), cytosol (lane 3), and mitochondria (lane 4). Isolated mitochondria were further treated with proteinase K in the absence (lane 5) or presence (lane 6) of Triton X-100. Fractions were analysed using western blotting for the indicated topoisomerases and using markers for the nucleus (H3), mitochondrial matrix (MRPS27 and TFAM) and mitochondrial outer membrane (TOM20).

(D) Extended exposures of cellular fractions of K562 cells as shown in Figure S1C. **(E)** Extended exposures of western blots from submitochondrial fractions of K562 cells as shown in Figure 1D, for the four topoisomerases that did not show evidence of mitochondrial localisation in shorter exposures (TOP1, TOP2A, TOP2B, and TOP3B). **(F)** Comparison of expression levels of TOP2A in whole cell extract samples of mouse liver and spleen using western blotting. H3 is used as a loading control. **(G)** Control for thermal degradation of proteins under conditions used for proteinase K treatment. Identical samples of WCE (lanes 1-2), N/D (lanes 3-4) or cytosol (lanes 5-6) were incubated on ice (lanes 1, 3 and 5) or at 37°C (lanes 2, 4 and 6) for 30 min and then western blotted with the indicated antibodies. **(H-I)** Validation of the specificity of topoisomerase antibodies used. **(H)** HeLa cells were transfected using a control siRNA (lane 1) or siRNAs specific to each of the six human topoisomerases (lanes 2-7). Total protein samples were analysed by western blotting using antibodies specific to each topoisomerase as indicated. β-actin is used as a loading control. **(I)** The specificity of the TOP1MT (1) antibody was assessed using crude mitochondrial extracts from untransfected, control siRNA-treated cells and TOP1MT siRNA-treated cells because of antibody sensitivity issues. *n.s. indicates a non-specific band.

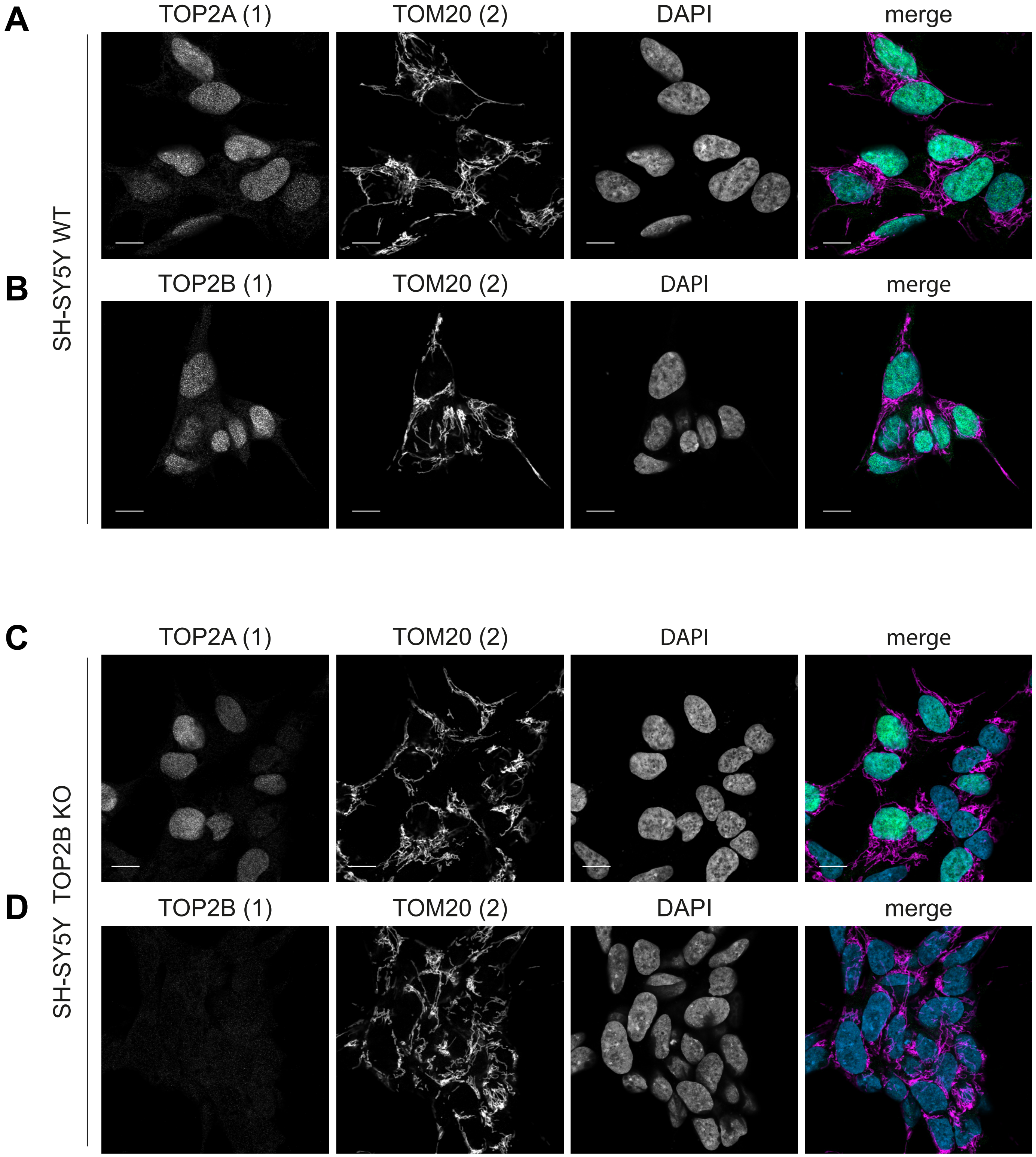
A
B
C
D
TOP2A (1)
TOM20 (2)
DAPI
merge
TOP2B (1)
SH-SY5Y WT
SH-SY5Y TOP2B KO

Figure S2. *Further localisation of TOP2 isoforms using confocal microscopy.*

(A-B) SH-SY5Y WT cells were stained for TOP2A **(A)** or TOP2B **(B)** with TOM20 used as a mitochondrial marker and DAPI as a nuclear marker. In the merged image, the topoisomerase is shown in green, TOM20 in magenta, and DAPI in blue. Scale bars represent 10 μm. **(C-D)** SH-SY5Y TOP2B knockout cells were stained for TOP2A **(C)** or TOP2B **(D)**, with controls as in (A-B). Images were taken using identical settings to allow for direct comparison. Scale bars represent 10 μm.

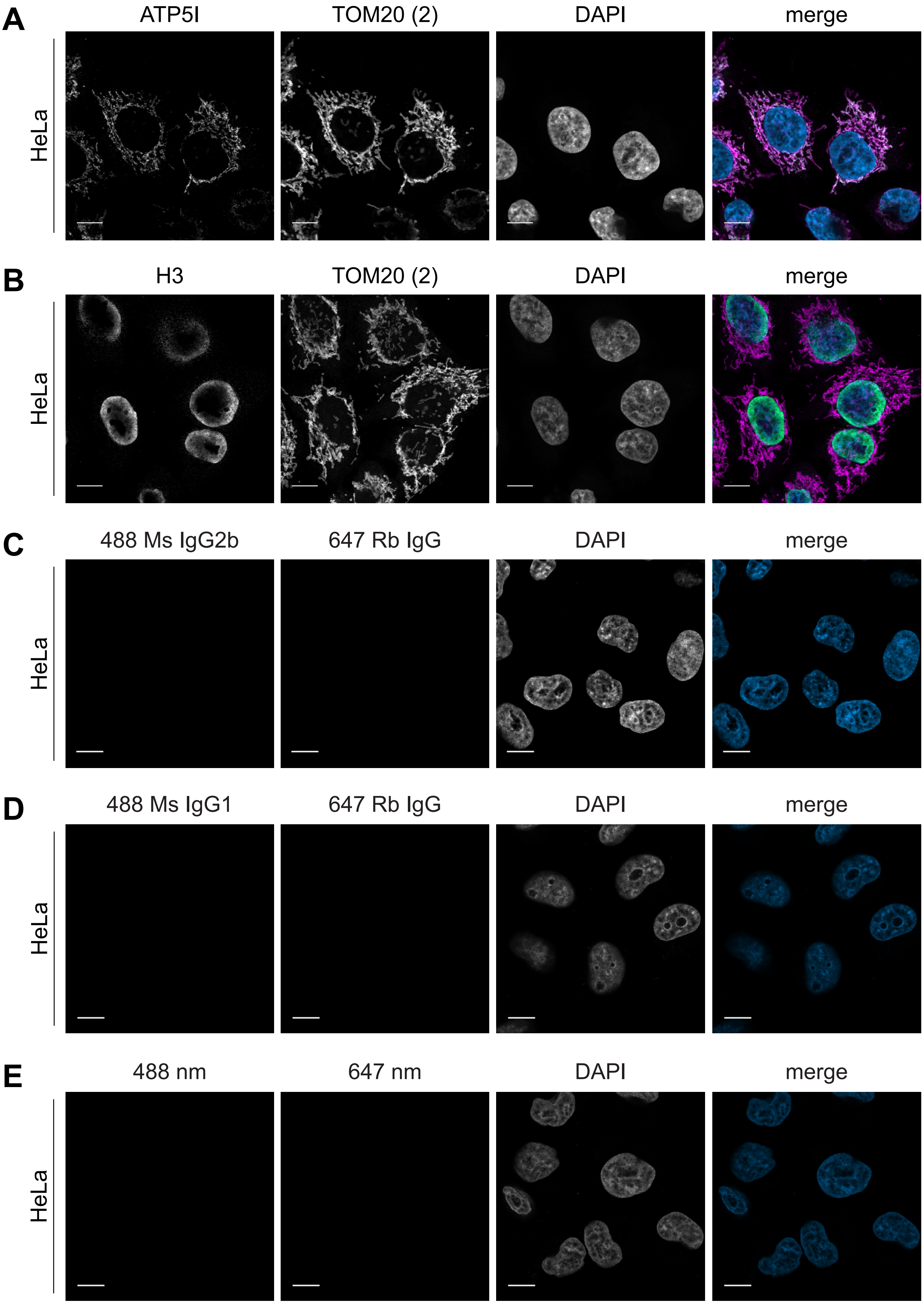
A
ATP5I
TOM20 (2)
DAPI
merge
HeLa
B
H3
TOM20 (2)
DAPI
merge
HeLa
C
488 Ms IgG2b
647 Rb IgG
DAPI
merge
HeLa
D
488 Ms IgG1
647 Rb IgG
DAPI
merge
HeLa
E
488 nm
647 nm
DAPI
merge
HeLa

Figure S3. *Controls for localisation experiments using microscopy.*

(A-B) Positive controls for protein co-localisation using microscopy. HeLa cells were stained using antibodies specific to ATP5I as a positive control for mitochondrial localisation **(A)** or H3 as a positive control for nuclear localisation **(B)**. TOM20 and DAPI are used as mitochondrial and nuclear markers, respectively, as in Figure 2. Scale bars represent 10 µm. **(C-D)** Airyscan images of HeLa cells incubated with only secondary antibodies as controls for antibody specificity. 488 Ms IgG2b, 647 Rb IgG, and 488 Ms IgG1 are the secondary antibodies corresponding to the primary antibodies used for TOP2A, TOM20 and TOP2B, respectively. Images were taken using identical settings to the corresponding images in Figure 2 to allow for direct comparison. In the merged image, the colours used for the channels corresponds to those in Figure 2 (TOP2A and TOP2B are shown in green, TOM20 in magenta and DAPI in blue). Scale bars represent 10 µm. **(E)** Images of untransfected HeLa cells at the wavelengths used for imaging of eGFP (488 nm) and TOM20 (647 nm) as controls for autofluorescence. Scale bars represent 10 µm.

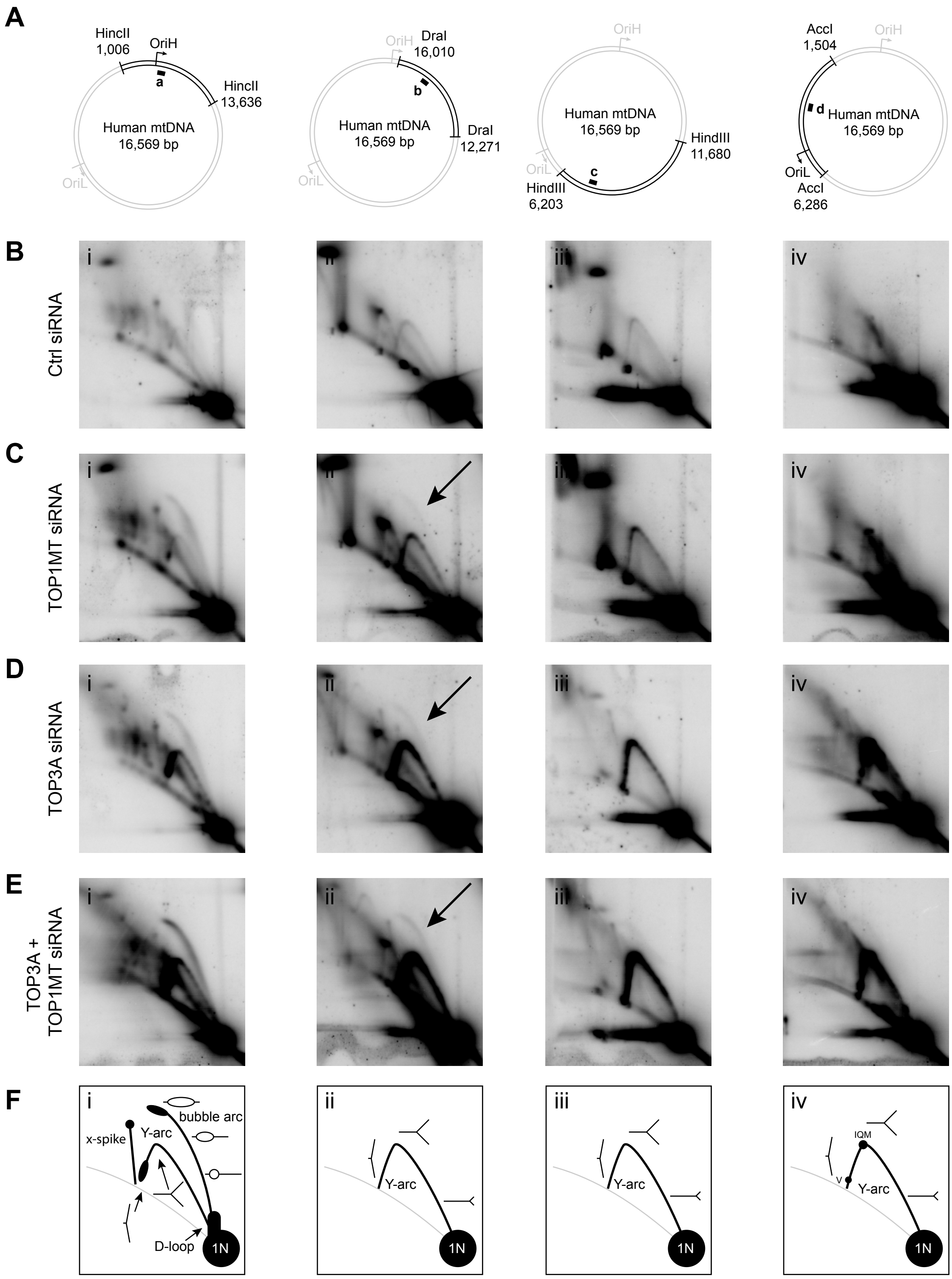
A
HincII 1,006
OriH
a
HincII 13,636
Human mtDNA 16,569 bp
OriL
OriH
DraI 16,010
b
Human mtDNA 16,569 bp
DraI 12,271
OriL
OriH
Human mtDNA 16,569 bp
HindIII 11,680
OriL
c
HindIII 6,203
AccI 1,504
OriH
d
Human mtDNA 16,569 bp
OriL
AccI 6,286
B
Ctrl siRNA
C
TOP1MT siRNA
D
TOP3A siRNA
E
TOP3A + TOP1MT siRNA
F
i
ii
iii
iv
bubble arc
x-spike
Y-arc
D-loop
1N
IQM
V

Figure S4. *Extended exposures of 2DNAGE blots.*

Extended exposures of 2DNAGE panels as in Figure 3. **(A)** mtDNA was restricted using the indicated enzymes. The black bar indicates the location of the probe. **(B-E)** 2DNAGE blots from control siRNA treated cells **(B)**, TOP1MT depleted cells **(C)**, TOP3A depleted cells **(D)**, and TOP3A+TOP1MT depleted cells **(E).** Bubble-arc products, indicative of replication initiation in DraI restriction fragments, are indicated with a black arrow in panels (C-E), panel ii. **(F)** Diagrams indicating prominent replication intermediates observed using 2DNAGE.

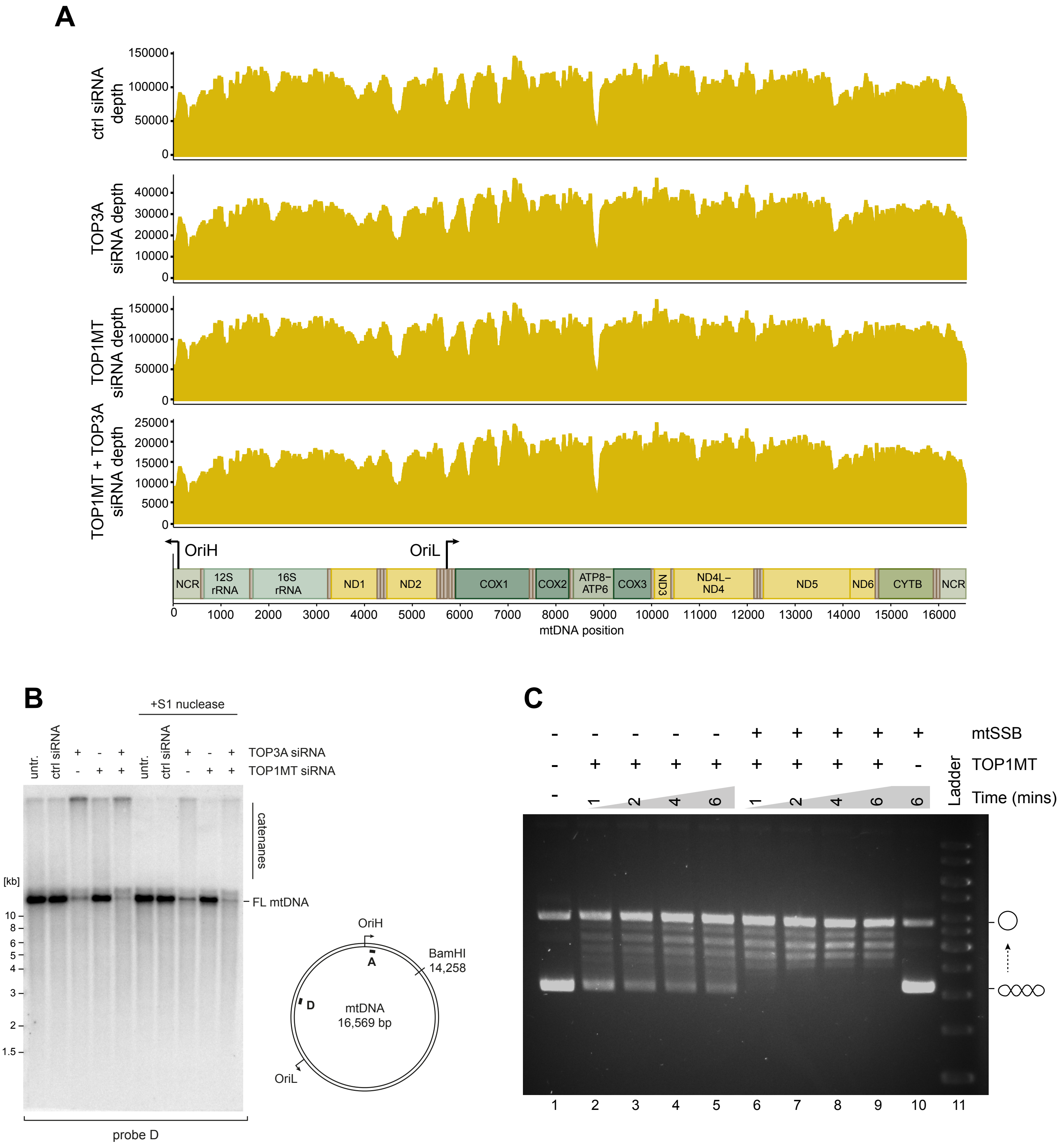
A
ctrl siRNA depth
TOP3A siRNA depth
TOP1MT siRNA depth
TOP1MT + TOP3A siRNA depth
150000
100000
50000
0
40000
30000
20000
10000
25000
20000
15000
10000
5000
OriH
OriL
NCR
12S rRNA
16S rRNA
ND1
ND2
COX1
COX2
ATP8–ATP6
COX3
ND3
ND4L–ND4
ND5
ND6
CYTB
NCR
0 1000 2000 3000 4000 5000 6000 7000 8000 9000 10000 11000 12000 13000 14000 15000 16000
mtDNA position
B
+S1 nuclease
untr.
ctrl siRNA
TOP3A siRNA
TOP1MT siRNA
catenanes
[kb]
FL mtDNA
10
8
6
5
4
3
2
1.5
probe D
OriH
A
BamHI 14,258
D
mtDNA 16,569 bp
OriL
C
mtSSB
TOP1MT
Time (mins)
Ladder
1 2 4 6 1 2 4 6 6
1 2 3 4 5 6 7 8 9 10 11

Figure S5. *Roles of TOP3A and TOP1MT in mtDNA replication.*

(A) Depth profiles for whole genome sequencing of HeLa cells following treatment with control siRNA, TOP3A siRNA, TOP1MT siRNA, or TOP3A+TOP1MT siRNA. The location of replication origins and mtDNA gene loci are indicated in the diagram below. **(B)** Southern blot of BamHI-linearised total DNA from topoisomerase-depleted cells as in Figure 4C, and hybridised with probe D in the mtDNA minor arc, as indicated in the diagram (right). **(C)** Time-course of the relaxation of negatively supercoiled pUC18 DNA by 25 nM recombinant TOP1MT in the absence (lanes 2-5) or presence (lanes 6-9) of 200 nM mtSSB. The migration of supercoiled and relaxed substrate DNA is indicated.

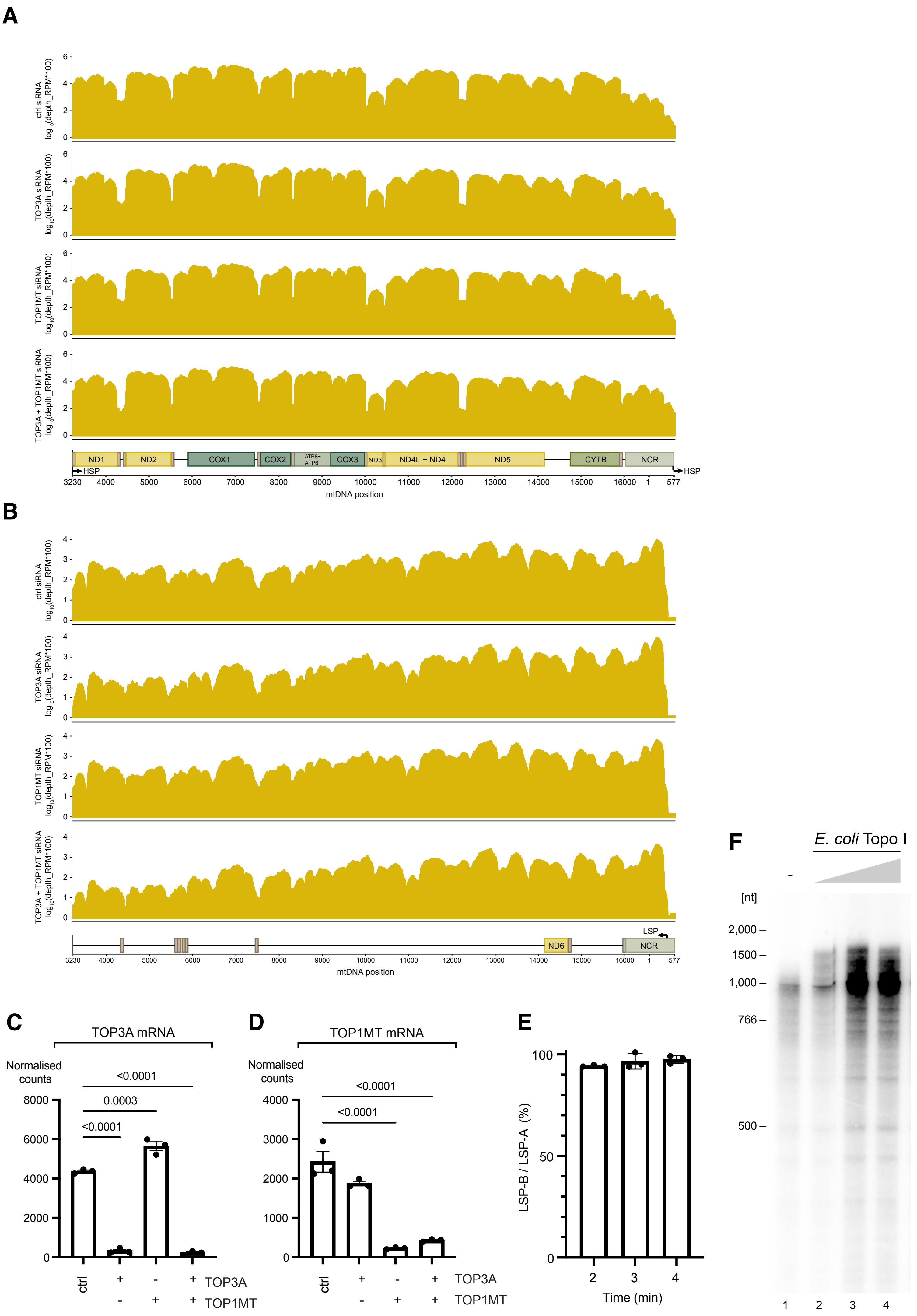

A
ctrl siRNA
log10(depth_RPM*100)
TOP3A siRNA
TOP1MT siRNA
TOP3A + TOP1MT siRNA
ND1
ND2
COX1
COX2
ATP8-ATP6
COX3
ND3
ND4L – ND4
ND5
CYTB
NCR
HSP
3230 4000 5000 6000 7000 8000 9000 10000 11000 12000 13000 14000 15000 16000 1 577
mtDNA position
B
ND6
LSP
C
TOP3A mRNA
Normalised counts
<0.0001
0.0003
<0.0001
8000 6000 4000 2000 0
ctrl
TOP3A
TOP1MT
D
TOP1MT mRNA
Normalised counts
<0.0001
<0.0001
4000 3000 2000 1000 0
E
LSP-B / LSP-A (%)
100 50 0
2 3 4
Time (min)
F
E. coli Topo I
[nt]
2,000 –
1500 –
1,000 –
766 –
500 –
1 2 3 4

Figure S6. *Roles of TOP3A and TOP1MT in mitochondrial transcription.*

(A-B) Normalised RNA-seq depth profiles from topoisomerase-depleted cells. Profiles are shown for HSP-derived transcripts **(A)** and LSP-derived transcripts **(B)**. Depth is expressed as $\log_{10}$-transformed reads per million (RPM)×100. Promoter locations and gene loci are shown in diagrams beneath the depth profiles. **(C-D)** Normalised counts of TOP3A **(C)** and TOP1MT **(D)** in the RNA-seq dataset of topoisomerase-depleted cells as in (A-B). n = 3, error bars represent ±SEM, p values are indicated from one-way ANOVA. **(E)** Quantification of the relative proportions of LSP-A and LSP-B transcription products as in Figure 6G. Error bars represent mean values ±SEM, n = 3. **(F)** *In vitro* transcription reactions using a dual-promoter template as in Figure 6H, with the addition of *E. coli* TopoI. Reactions contained POLRMT, TFAM, TFB2M and TEFM in the absence (lane 1) or presence (lanes 2-4) of TopoI (0.125, 0.25, or 0.5 U) as indicated.

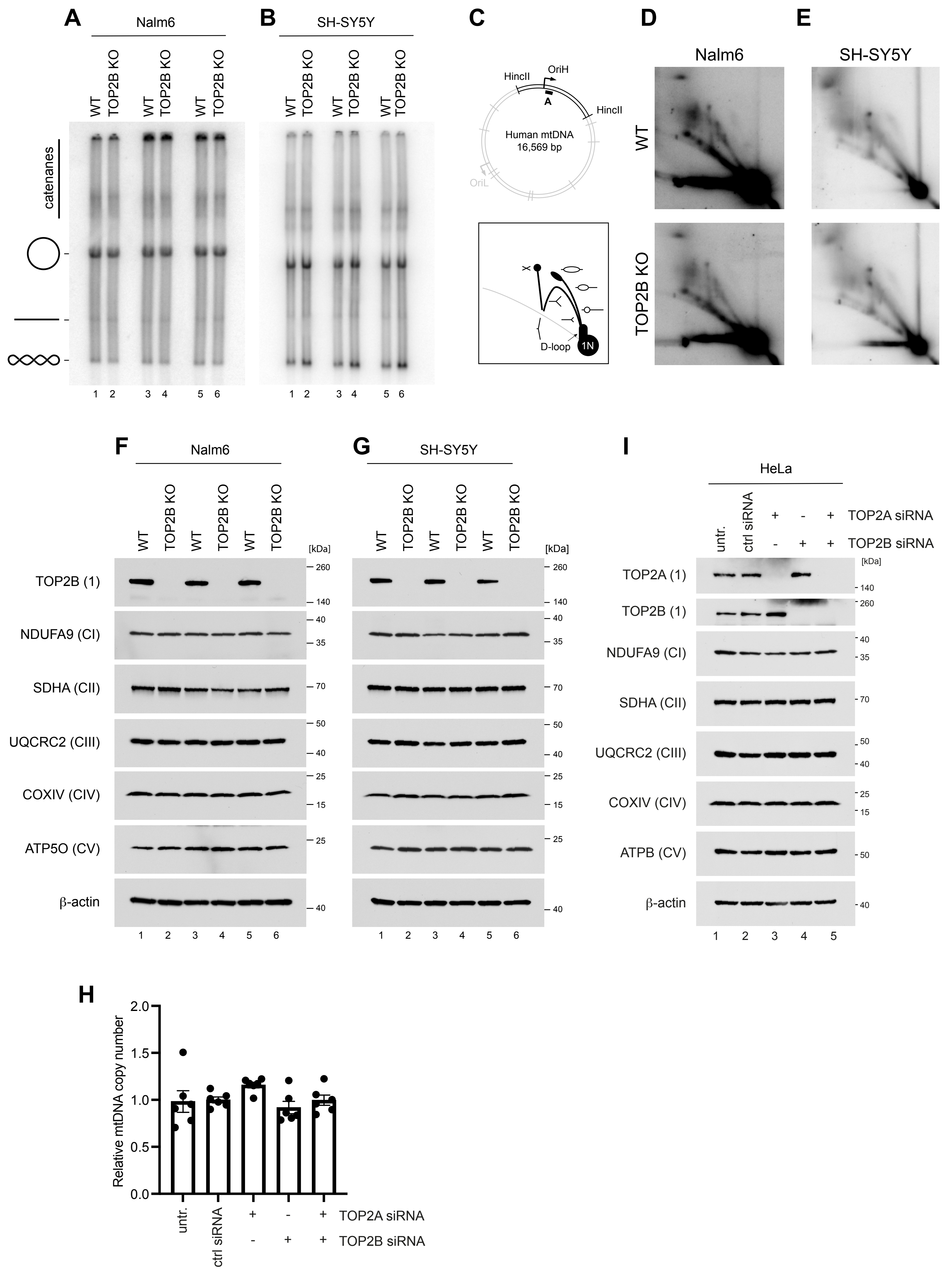
A
Nalm6
WT
TOP2B KO
catenanes
1 2 3 4 5 6
B
SH-SY5Y
C
HincII
OriH
A
HincII
Human mtDNA
16,569 bp
OriL
D-loop
1N
D
Nalm6
E
SH-SY5Y
WT
TOP2B KO
F
Nalm6
[kDa]
TOP2B (1)
NDUFA9 (CI)
SDHA (CII)
UQCRC2 (CIII)
COXIV (CIV)
ATP5O (CV)
β-actin
260
140
40
35
70
50
40
25
15
25
40
G
SH-SY5Y
I
HeLa
untr.
ctrl siRNA
TOP2A siRNA
TOP2B siRNA
TOP2A (1)
TOP2B (1)
ATPB (CV)
1 2 3 4 5
H
Relative mtDNA copy number
2.0
1.5
1.0
0.5
0.0

Figure S7. *Characterisation of TOP2B-knockout and TOP2A/TOP2B-depleted cells.*

(A-B) mtDNA topology following knockout of TOP2B in Nalm6 cells **(A)** and SH-SY5Y cells **(B)**. Uncut mtDNA was separated on agarose gels and Southern blotted using the mtDNA-specific probe A. The migration of open-circle form, linear, and supercoiled mtDNA is indicated. **(C-E)** Assessment of mtDNA replication patterns in WT and TOP2B knockout Nalm6 cells **(D)** and SH-SY5Y cells **(E)** assessed using 2DNAGE. The diagram **(C)** indicates the location of the analysed HincII restriction fragment and probe (top panel) and schematic of observed mtDNA replication intermediates (bottom panel). **(F-G)** Levels of respiratory complex proteins following knockout of TOP2B in Nalm6 **(F)** and SH-SY5Y **(G)** cells assessed using western blotting. One marker protein is used for each of complexes I-V (CI-CV), and β-actin is used as a loading control. Replicates represent separate protein extractions from sequential passages of each cell line. **(H)** mtDNA copy number in TOP2A and TOP2B-depleted HeLa cells measured using qPCR. Copy number is expressed as the level of an mtDNA-specific amplicon (ND1) normalised to the level of a nuclear amplicon (B2M) normalised to the control siRNA sample. Plot shows mean values, $n = 6$, error bars represent ±SEM. No significant differences are present using one-way ANOVA. **(I)** Levels of TOP2A, TOP2B, and respiratory complex proteins following depletion of TOP2A and TOP2B as in (H) assessed using western blotting. One marker protein is used for each of complexes I-V (CI-CV), and β-actin is used as a loading control.

Table S1: siRNA oligonucleotides used in this study

Target	Ambion assay ID	Sense sequence (5'-3')	PubChem SID
TOP1	s14304	GGAUGAUCUUUUUGAUAGAtt	160718244
TOP1MT	s42017	CCCUGUAUUUCAUCGAUAAtt	160755427
TOP2A	s14307	GGAUUCUGCUAGUCCACGAtt	160718247
TOP2B	s106	CGAUUAAGUUAUUACGGUUtt	160714417
TOP3A	s14310	CGGCUUGCCUAGUUCUCUAtt	160718251
TOP3B	s17097	CCACCCUUCCGAGACAUGAtt	160721182

Table S2: Antibodies used in this study

Primary antibodies

Target	Supplier	Cat. no.	RRID
AIF	Cell Signaling Technology	4642	RRID:AB_2224542
ATP5I	Proteintech	16483-1-AP	RRID:AB_2062052
ATP5O	Abcam	ab110276	RRID:AB_10887942
ATPB	Abcam	ab14730	RRID:AB_301438
Beta-actin	Abcam	ab6276	RRID:AB_2223210
BrdU	Merck Millipore	11170376001	RRID:AB_514483
COXIV	Abcam	ab110261	RRID:AB_10862101
H3	Abcam	ab1791	RRID:AB_302613
MRPS27	Proteintech	17280-1-AP	RRID:AB_2180510
NDUFA9	Abcam	ab14713	RRID:AB_301431
SDHA	Abcam	ab14715	RRID:AB_301433
TFAM (1)	Santa Cruz Biotechnology	sc-376672	RRID:AB_11150497
TFAM (2)	Abcam	ab131607	RRID:AB_11154693
TOM20 (1)	Abcam	ab78547	RRID:AB_2043078
TOM20 (2)	Abcam	ab186734	RRID:AB_2716623
TOP1	BD Biosciences	556597	RRID:AB_396474
TOP1MT (1)	Sigma Aldrich	HPA001915	RRID:AB_1080331
TOP1MT (2)	Proteintech	16540-1-AP	RRID:AB_2878274
TOP2A (1)	Santa Cruz Biotechnology	sc-166934	RRID:AB_10611755
TOP2A (2)	Abcam	ab52934	RRID:AB_883143
TOP2B (1)	Santa Cruz Biotechnology	sc-25330	RRID:AB_628384
TOP2B (2)	N/A, Rb pAb, TOP2B 1263-1621	N/A	N/A
TOP2B (3)	N/A, Rb pAb, TOP2B 46-1621	N/A	N/A
TOP2B (4)	Novus	NB100-40842	RRID:AB_792364
TOP3A	Proteintech	14525-1-AP	RRID:AB_2205881
TOP3B	Abcam	ab183520	N/A
UQCRC2	Abcam	ab14745	RRID:AB_2213640
VDAC	Abcam	ab14734	RRID:AB_443084

Secondary antibodies

Name	Supplier	Cat. no.	RRID
Alexa Fluor 488 goat anti-mouse IgG2b	Life Technologies	A21141	RRID:AB_141626
Alexa Fluor 488 goat anti-mouse IgG1	Life Technologies	A21121	RRID:AB_2535764
Alexa Fluor 594 goat anti-mouse IgG	Life Technologies	A11032	RRID:AB_2534091
Alexa Fluor 647 goat anti-rabbit IgG	Life Technologies	A21244	RRID:AB_2535812
Atto 647N goat anti-rabbit IgG	Sigma-Aldrich	40839	RRID:AB_1137669
Rabbit anti-mouse HRP	Agilent	P0260	RRID:AB_2636929
Swine anti-rabbit HRP	Agilent	P0217	RRID:AB_2728719

Table S3: Oligonucleotides for qPCR and Southern blotting

qPCR primers and probes

Name	Sequence (5'-3')
B2M Forward	CCAGCAGAGAATGGAAAGTCAA
B2M Reverse	TCTCTCTCCATTCTTCAGTAAGTCAACT
B2M Probe	6-FAM-ATGTGTCTGGGTTTCATCCATCCGACA-MGB
ND1 Forward	CCCTAAAACCCGCCACATCT
ND1 Reverse	GAGCGATGGTGAGAGCTAAGGT
ND1 Probe	VIC-CCATCACCCTCTACATCACCGCCC-MGB

Oligonucleotides for Southern blotting probes

Name	Sequence (5'-3')
Probe A Forward	CTCACCCACTAGGATACCAAC
Probe A Reverse	GATACTGCGACATAGGGTGC
Probe B Forward	CCACTCATTCATCGACCTCCC
Probe B Reverse	CCTAGGGGGTTGTTTGATCCC
Probe C Forward	GGCACATGCAGCGCAAGTAGG
Probe C Reverse	GACCGTAGTATACCCCCGGTC
Probe D Forward	GCCACATCTACCATCACCCTC
Probe D Reverse	TAGAGTTCAGGGGAGAGTGCG

Oligonucleotides for northern blotting probes

Name	Sequence (5'-3')
12S Forward	CAAAACGCTTAGCCTAGCC
12S Reverse	TAGCCCATTTCTTGCCACC
16S Forward	CACCCACTACCTAAAAAATCCC
16S Reverse	GTCTTGCTGTGTTATGCCC
COI Forward	ACTCTTACCTCCCTCTCTCC
COI Reverse	TCAGCTAAATACTTTGACGCC
COII Forward	TCTACAAGACGCTACTTCCC
COII Reverse	ACAGATTTCAGAGCATTGACC
COIII Forward	CCTACTAACCAACACACTAACC
COIII Reverse	ACCACATCTACAAAATGCCAG
CYTB Forward	CATCAATCGCCCACATCAC
CYTB Reverse	AGGAAGAGAAGTAAGCCGAG
5.8S rRNA	CAAGTGCGTTCGAAGTGTCGATGAT
